# Design of multivalent-epitope vaccine models directed toward the world’s population against *HIV-Gag* polyprotein: Reverse vaccinology and immunoinformatics

**DOI:** 10.1371/journal.pone.0306559

**Published:** 2024-09-27

**Authors:** Ava Hashempour, Nastaran Khodadad, Peyman Bemani, Younes Ghasemi, Shokufeh Akbarinia, Reza Bordbari, Amir Hossein Tabatabaei, Shahab Falahi

**Affiliations:** 1 HIV/AIDS Research Center, Institute of Health, Shiraz University of Medical Sciences, Shiraz, Iran; 2 Department of Immunology, School of Medicine, Isfahan University of Medical Sciences, Isfahan, Iran; 3 Department of Pharmaceutical Biotechnology, School of Pharmacy, Shiraz University of Medical Sciences, Shiraz, Iran; 4 Zoonotic Diseases Research Center, Ilam University of Medical Sciences, Ilam, Iran; National Institute of Biologicals (NIB), Ministry of Health & Family Welfare, Government of India, INDIA

## Abstract

Significant progress has been made in *HIV-1* research; however, researchers have not yet achieved the objective of eradicating *HIV-1* infection. Accordingly, in this study, eucaryotic and procaryotic in silico vaccines were developed for *HIV-Gag* polyproteins from 100 major *HIV* subtypes and CRFs using immunoinformatic techniques to simulate immune responses in mice and humans. The epitopes located in the conserved domains of the *Gag* polyprotein were evaluated for allergenicity, antigenicity, immunogenicity, toxicity, homology, topology, and IFN-γ induction. Adjuvants, linkers, CTLs, HTLs, and BCL epitopes were incorporated into the vaccine models. Strong binding affinities were detected between HLA/MHC alleles, TLR-2, TLR-3, TLR-4, TLR-7, and TLR-9, and vaccine models. Immunological simulation showed that innate and adaptive immune cells elicited active and consistent responses. The human vaccine model was matched with approximately 93.91% of the human population. The strong binding of the vaccine to MHC/HLA and TLR molecules was confirmed through molecular dynamic stimulation. Codon optimization ensured the successful translation of the designed constructs into human cells and *E*. *coli* hosts. We believe that the *HIV-1 Gag* vaccine formulated in our research can reduce the challenges faced in developing an *HIV-1* vaccine. Nevertheless, experimental verification is necessary to confirm the effectiveness of these vaccines in these models.

## 1. Introduction

Despite a global reduction in deaths caused by harmful pathogens, infectious diseases continue to impose significant catastrophes on human civilization worldwide [1–5]. Significant advances have been made in the treatment of infections; however, the burdens of sickness and death continue to increase substantially [6–8]. The implementation of new detection techniques, vaccines, medications, and improved hygiene has decreased the occurrence of infections and related fatalities [9–11]; nonetheless, continuing to invest in existing strategies to explore innovative therapeutic approaches is crucial [12–15]. For example, various studies have been conducted to find effective treatments or potential vaccine candidates to either eliminate or control the global health crisis caused by human immunodeficiency virus (*HIV)-1*, which causes acquired immunodeficiency syndrome (AIDS). Antiretroviral therapy (ART) decreases the transmission of *HIV* and slows the transmission of AIDS, leading to prolonged life expectancy [16]. Nevertheless, ARTs do not eradicate *HIV* from the body, necessitating the development of a potent vaccine to overcome numerous obstacles in the suppression of *HIV* infection.

Various vaccine techniques are employed to control *HIV* infection and AIDs, among which the conventional methods are old strategies that utilize either weakened or inactivated *HIV* [17]. At present, an effective method for vaccine development involves the use of advanced bioinformatic techniques to examine and predict virological and immunological results [18–20]. Multiepitope vaccines targeting several human tumor viruses have gained significant global attention, as exemplified by increased interest worldwide [21]. In addition, bioinformatics methods possess numerous advantages, such as effectiveness in preclinical trials, safety, target specificity, and simplicity of production. In other words, such valuable techniques employ computational methods such as molecular docking and molecular dynamics (MD) simulations to analyze vast quantities of biomedical data produced by different software programs, aiming to identify potential vaccine targets [22]. However, various challenges hinder the development of an ideal vaccine against *HIV*. These include 1) multiple funding issues associated with vaccine development; 2) the rapid mutation rate of the *HIV* genome due to replication, resulting in the emergence of new subtypes, circulating recombinant forms (CRFs), and strains; 3) the absence of a suitable animal model for studying *HIV* behaviors; 4) the establishment of a latent reservoir by *HIV-1* [23], the diverse modes of *HIV* transmission to humans; and [24] the inability of current vaccines to stimulate both humoral and cellular responses, as well as their failure to produce broadly neutralizing antibodies [25]. This is why the *HIV* vaccine fails to generate an effective immune response in the global human population. For instance, one of the highly effective vaccines developed, RV144, only leads to 31.2% protection against *HIV* [26]. According to previous findings, the *Gag* gene contains numerous highly immunogenic protective epitopes, making such important polyproteins key targets for cytotoxic T lymphocytes (CTLs) involved in the control of *HIV* replication and suppression [27]. For example, in the AIDS Clinical Trials Group (ACTG), patients with *HIV-1* infection were vaccinated with rAd5 *HIV-1 Gag*, which resulted in an increase in the activity of *HIV*-specific CD4 and CD8 cells [28]. Furthermore, administering DNA vaccines containing identical epitopes found in *SIV* p27^Gag^ to the *SIV*/macaque model resulted in a robust immune response [29].

This is the first study to suggest in silico vaccine constructs for *Gag-HIV* polyproteins using bioinformatics software. Another novelty of this report is that the suggested eukaryotic and prokaryotic models of vaccines are compatible with the main *HIV* subtypes and CRFs and induce both cellular and humoral immune responses in computer-based immune responses in mouse and human hosts. To achieve this goal, we obtained a total of one hundred full-length *HIV-1* sequences of the major *HIV* subtypes and CRFs from the Los Alamos National Laboratory (LANL) database. Subsequently, consensus sequences of the *Gag* polyproteins were generated. Multiple servers have been used to predict and evaluate thousands of CTL, helper T lymphocyte (HTL), and B-cell (BCL) epitopes based on various distinctive criteria, including topology, antigenicity, allergenicity, homology, population coverage, immunogenicity, toxicity, and induction of interferon-gamma (IFN-γ). Among the thousands of predicted epitopes, only those located in the conserved domains of the Gag polyprotein were considered candidates for inclusion in the vaccine construct sequence. The final sequence of the vaccine constructs was evaluated for structural characteristics, including secondary and tertiary structures, and molecular dynamics and molecular docking studies were conducted on Toll-like receptors (TLRs) 2, 3, 4, 7, and 9, which confirmed the long-term effectiveness of the vaccine model. To stimulate appropriate proinflammatory cytokines, such as IFN-ɣ, and the innate response through TLR docking, suitable adjuvants, including the C-terminal invasin sequence of *Yersinia*, beta defensin-3, and the pan-HLA DR-binding epitope (PADRE), were incorporated into the vaccine construct. Finally, codon-optimized vaccine sequence constructs were cloned and inserted into the pET-30a (+) plasmid and pcDNA3.1 (+) using SnapGene software to efficiently express the vaccine constructs in bacteria and human hosts, respectively.

## 2. Materials and methods

### 2.1. Data retrieval and sequence alignment

Up to 4th June 2023, one hundred full-length *HIV* genomes of the most common *HIV* subtypes and CRFs submitted to the LANL *HIV* sequence database were B, C, A, AE, D, G, F, AG, BC, O, BF, and CRF35-AD. Using CLC-sequence Viewer Software (version CLC Genomics Workbench 20) [1], *GAG* amino acid sequences were obtained from the mentioned subtypes and CRFs and aligned to generate the consensus sequence that was considered for further analysis. Intersequence homology was examined by CLC-sequence Viewer considering the following parameters: gap opening cost: 10 and gap extension cost: 1.0.

### 2.2. Conserved domain analysis

To design a more effective vaccine construct, NCBI CDD-BLAST was used to define the conserved domains of the *Gag* sequence to select the most promising epitopes from the mentioned domains.

### 2.3. Prediction of biophysical and biochemical features

The ProtParam tool was used for the purpose of analyzing a variety of physicochemical properties of the consensus *Gag* sequence. These properties include the number of amino acids, molecular weight, theoretical isoelectric point (pI), estimated half-life (both in vitro and in vivo), as determined by the N-end rule, aliphatic index, and grand average of hydropathy (GRAVY) index of the vaccine construct [30–32].

### 2.4. Prediction and selection of linear BCL epitopes

The BCL epitope, also known as the antigenic determinant, is precisely defined as the specific portion of the antigen that is recognized by the BCL receptor or its soluble form, namely, antibodies, which are secreted following the activation of BCLs [33–35]. BCL epitopes can be categorized as either conformational, which are also referred to as discontinuous, or linear, also known as continuous. In the case of proteins, conformational BCL epitopes encompass residues that are not sequential in the primary structure but are in close proximity within the three-dimensional structure of the antigen [35, 36]. Conversely, linear BCL epitopes consist of consecutive amino acid residues. These BCL epitopes can be identified by antibodies independently of the surrounding protein context and can be used as a substitute for the entire protein in antibody production [35]. Multiple approaches and techniques are available for the prediction of linear BCL epitopes [35–40]. Some of these techniques employ amino acid propensity scales that illustrate the physicochemical characteristics of BCL epitopes. These techniques include ABCpred, Bepipred, Emini, Karplus, and Parker [41].

#### 2.4.1. ABCpred

The ABCpred server was used to predict the linear BCL epitopes specific to the humoral immune response of the aforementioned Gag protein. This prediction was accomplished through the utilization of an artificial neural network, which is the basis of the algorithm employed by the server. The neural network consists of 700 BCl epitopes and 700 non-BCL epitopes, each with a maximum length of 20 residues, for training and testing purposes [42, 43]. The server has demonstrated an accuracy of 65.93% using this recurrent neural network. Consequently, the FASTA sequence of the Gag protein was used to predict linear BCL epitopes with a threshold of 0.51 and a window length of 10 [44].

#### 2.4.2. BepiPred-2.0

The BepiPred-2.0 method relies on a random forest algorithm that has been trained on epitopes annotated from antibody-antigen protein structures [45]. This novel approach has been shown to outperform other available tools for predicting epitopes based on sequence. The superiority of this method was demonstrated by evaluating epitope data derived from solved 3D structures, as well as a large collection of linear epitopes obtained from the Immune Epitope Database (IEDB). The results of the method are presented in a user-friendly and informative manner, catering to both computer-savvy and nonexpert users [46].

#### 2.4.3. Emini surface accessibility

The Emini surface accessibility prediction method utilizes the calculation of surface probability to increase confidence in provisional alignment for sequence comparison [47]. This prediction is based on the method proposed by Garnier et al. [48] and the Chou and Fasman method [49]. Furthermore, this method assumes the absence of significant internal deletions or insertions. The IEDB database was used to predict surface accessibility using the default threshold value for each protein [50].

#### 2.4.4. Karplus flexibility

The Karplus flexibility scale method constructs a flexibility scale based on the mobility of protein segments. This scale is derived from the known temperature B factors of the α-carbons of 31 proteins with known structures. The calculation based on this flexibility scale is similar to the classical calculation, with the exception that the center is the first amino acid of the window length of six amino acids, and three scales are used to describe flexibility instead of just one [51].

#### 2.4.5. Parker hydrophilicity

The Parker hydrophilicity prediction method constructs a hydrophilic scale based on the retention times of peptides during high-performance liquid chromatography (HPLC) on a reversed-phase column. The epitope region was analyzed using a window of seven residues [41]. Finally, the epitopes were screened through the following filters:

### 2.5. Selection of top epitopes

Thousands of epitopes were suggested for the *Gag* sequence by different methods; however, the selection of high-quality epitopes was based on the combination of filters used in this study.

#### 2.5.1. Antigenicity evaluation

Epitopes that bind to immune cells with strong binding affinity exhibit high antigenicity [52]. The VaxiJen v2.0 server (Table 1g) with a threshold level of 0.4 was utilized to assess the antigenicity of an epitope [52], and any epitopes that did not meet this requirement were discarded. VaxiJen’s prognostications regarding antigenicity are based on the application of autocross covariance transformations to protein sequences, resulting in standardized vectors of principal amino acid characteristics [53, 54].

**Table 1:** List of links used in this study.

Table	Software	URL	Function
**1a**	LANL	www.hiv.lanl.gov	HIV sequence database
**1b**	NCBI CDD-BLAST	https://www.ncbi.nlm.nih.gov/Structure/cdd/wrpsb.cgi	Conserved domain analysis
**1c**	BcePred	http://ailab-projects1.ist.psu.edu:8080/bcpred/predict.html	Linear BCL epitopes
	Bepipred	http://www.cbs.dtu.dk/services/BepiPred/	
	Emini	http://tools.iedb.org/bcell/	
	karplus	http://tools.iedb.org/bcell/	
	Parker	http://tools.iedb.org/bcell/	
**1d**	ProtParam	https://web.expasy.org/protparam/	Physico-chemical properties
**1e**	SOPMA	https://npsa-prabi.ibcp.fr/NPSA/npsa_sopma.html	Secondary structure prediction
	mRNAfold	http://rna.tbi.univie.ac.at/cgi-bin/RNAWebSuite/RNAfold.cgi	
**1f**	I-TASSER	https://zhanglab.ccmb.med.umich.edu/I-TASSER/	Tertiary structure prediction
	GalaxyRefine	http://galaxy.seoklab.org/cgi-bin/submit.cgi?type=REFINE	Protein refinement
	ProSA-web	https://prosa.services.came.sbg.ac.at/prosa.php	Protein model validation
	RAMPAGE	http://www.ebi.ac.uk/thornton-srv/databases/pdbsum/Generate.html	
	ERRAT	https://servicesn.mbi.ucla.edu/ERRAT/	
		https://saves.mbi.ucla.edu/	
	Qmean	https://swissmodel.expasy.org/qmean/	
**1g**	VaxiJen v2.0	https://www.ddg-pharmfac.net/vaxijen/VaxiJen/VaxiJen.html	Antigenicity evaluation
	ToxinPred	https://webs.iiitd.edu.in/raghava/toxinpred/multi_submit.php	Identification of toxic epitopes
	AllerTOP V2.0	http://www.ddg-pharmfac.net/AllerTOP/	Allergen epitope prediction
	AllergenFP v.1.0	http://ddg-pharmfac.net/AllergenFP	
	Peptide match server	https://research.bioinformatics.udel.edu/peptidematch/index.jsp	Detection of the nonhomologous epitope to human proteome
	Topology	https://dtu.biolib.com/DeepTMHMM	Transmembrane prediction
	IFNepitope	https://webs.iiitd.edu.in/raghava/ifnepitope/predict.php	IFNγ release prediction
	Class I Immunogenicity	http://tools.iedb.org/immunogenicity/	Immunogenicity prediction
	CSM-Toxin webserver	https://biosig.lab.uq.edu.au/csm_toxin/predict	Identification of toxicity of sequence
**1h**	MHCII	http://tools.iedb.org/mhcii	HTL epitope prediction
**1i**	NetCTL	https://services.healthtech.dtu.dk/services/NetCTL-1.2/	CTL epitope prediction
	NetMHC-4.0/	https://services.healthtech.dtu.dk/services/NetMHC-4.0/	
	HLA class-I binding	http://tools.immuneepitope.org/mhci/	
**1j**	Ellipro	http://tools.iedb.org/ellipro/	Discontinuous BCL epitopes
**1k**	VectorBuilder server	https://en.vectorbuilder.com/tool/codon-optimization.html	Codon optimization
**1l**	PEP-FOLD 4.0 server	https://mobyle2.rpbs.univ-paris-diderot.fr/cgi-bin/portal.py#forms::PEP-FOLD4	Creation of pdb
	Protein database website	https://www.rcsb.org/	
	RCSB database	https://www.rcsb.org	
**1m**	ClusPro 2.0 online server	https://cluspro.bu.edu/login.php	Molecular docking and dynamic stimulation
	GROMACS software	http://www.mdtutorials.com/gmx/lysozyme/	
**1n**	C-IMMSIM	https://kraken.iac.rm.cnr.it/C-IMMSIM/index.php?page=1	Immune stimulation
**1o**	HighCharts	https://www.highcharts.com/chat/gpt/	Population coverage (generation of world map)

#### 2.5.2. Prediction of toxicity

The ToxinPred webserver and CSM-Toxin webserver (Table 1g) were used to identify the toxicity of the epitopes and choose nontoxic epitopes [55]. Based on quantitative matrices and machine learning, the toxicity of the epitopes was predicted by CSM-Toxin [56] and ToxinPred with an accuracy of 94.50% [53]. This server detects certain amino acid residues that are located at specific positions on toxic peptides.

#### 2.5.3. Allergen prediction

The epitope candidates should not be allergenic; therefore, the potential allergenicity of the vaccine was assessed using and AllerTOP V2.0 (Table 1g) [57]. These server employs an autocovariance (ACC) transformation technique to convert protein sequences into uniform vectors of equal length. Using the K-nearest neighbor algorithm, proteins were classified based on a training set comprising 2210 allergens and 2210 nonallergens from diverse species [44] also AllergenFP v.1.0 [58] was the other webserver used for predicting the allergenicity.

#### 2.5.4. Analysis of homology

With the use of the peptide match server (Table 1g), peptides can be matched with the complete UniProtKB human proteome in a timely and precise manner. To prevent the risk of host autoimmune diseases and cross-reactions, each epitope was subjected to additional scrutiny through the peptide match server [59].

#### 2.5.5. Topology forecasting

Because the majority of amino acids present in the transmembrane region of proteins are hydrophobic, determining the transmembrane sequence of vaccines is crucial. To accomplish this goal, the DeepTMHMM server (Table 1g), which is considered to be the most comprehensive and best-performing method for predicting the topology of both alpha-helical and beta-barrel transmembrane proteins, was utilized to identify the transmembrane domains of the vaccine [60].

#### 2.5.6. Predicting IFNγ-inducing epitopes

Based on the amino acid composition, positional conservation of residues, and peptide length, the IFNepitope server (Table 1g), which employs motif-based prediction and a hybrid approach of both machine learning methods, predicts the ability of the epitopes to release the IFNγ cytokine [53].

#### 2.5.7. Immunogenicity prediction

The immunogenicity of the epitopes was determined by the IEDB Class I Immunogenicity Tool (Table 1g), which predicts the immunogenicity of the epitope based on nonanchor position amino acids that play a less significant role in the binding affinity of the peptide [53].

### 2.6. Prediction and selection of the HTL epitope

With the utilization of the IEDB major histocompatibility complex (MHC) II binding server, a set of 15-mer epitopes was identified for mouse alleles (H2-IAb, H2-IAd, H2-IEd) based on a selected percentile rank of less than 10. To further refine the selection, various programs, such as VaxiJen v2.0, Immunogenicity, AllerTOP v.2.0, ToxinPred, TMHMM-2.0, topology, and PIR peptide matching, were used for filtration purposes. Subsequently, the qualified epitopes were subjected to screening through the IFNepitope webserver, which specializes in the identification of epitopes that induce IFN-γ production. Furthermore, these selected epitopes were cross-validated with the IEDB class II immunogenicity server, which is specifically designed for human leukocyte antigen (HLA) class II binding analysis [61]. Finally, the epitopes that exhibited the highest HLA class-II coverage were prioritized for population coverage assessment.

### 2.7. Prediction and selection of the CTL epitope

The IEDB MHC I server was used to forecast CTL epitopes. The main requirement was the IEDB Recommendation 2020.09 (NetMHCpanEL4.1), while the additional requirement encompassed epitopes of all human HLA alleles. Epitopes possessing a percentile level less than 0.5 were deemed suitable for the ensuing analysis stage. Subsequently, the Class I immunogenicity server was used to scrutinize the immunogenicity of these CTL epitopes, and only those epitopes with percentile levels less than 0.5 and immune scores greater than 1 were selected for further examination. To conclude, the VaxiJen v2.0 server was utilized to forecast antigenicity, employing a threshold value of 0.4, as referenced in a prior investigation [62]. Ultimately, CTL epitopes displaying immunological advantages were harnessed in the fabrication of a multiepitope vaccine.

### 2.8. Population coverage

The variety of HLA molecules is truly impressive, as more than a thousand unique allelic variants have been identified. The frequencies of these HLA alleles display notable differences among diverse ethnic groups. Therefore, the selection of different epitopes with varied HLA-binding specificities is of utmost importance when undertaking the design and development of T-cell epitope-based diagnostics or vaccines. This methodology will lead to increased coverage of the patient population, guaranteeing efficacy and inclusiveness [63]. The main purpose of the coverage analysis was to evaluate the suitability of selected epitope candidates for extensive populations [64]. A list of selected epitopes (CTL and HTL) and the corresponding alleles is provided in S1 and S2 Tables. A thorough examination was performed, wherein we collected data concerning population coverage, average epitope hits, and the proportion of the population constituting 90% (PC90), categorized by country and geographical region. Our findings included 115 countries and 16 geographically different regions [65].

### 2.9. Construction of a multiepitope subunit vaccine

In the final vaccine construct, the qualified epitopes of the Gag polyprotein that were located in conserved domains overlapped with other epitopes and were located in the conserved domains of the Gag polyprotein were included in the sequence of the vaccine construct. To protect the construct from degradation [66] and stimulate robust immune reactions, especially mucosal immune responses toward *HIV* [67], the beta defensin-3 adjuvant (GIINTLQKYYCRVRGGRCAVLSCLPKEEQIGKCSTRGRKCCRRKK) was incorporated at the N-terminus of the vaccine sequence. To overcome issues caused by highly polymorphic HLA class II alleles, the EAAAK linker and universal PADRE (AKFVAAWTLKAAA) were added [68] after the addition of the beta defensin-3 adjuvant to the vaccine construct. CTL epitopes were joined using GGGS linkers, HTL epitopes were joined using GPGPG linkers, and BCL epitopes were joined using KK linkers. The C-terminal invasin sequence of Yersinia (TAKSKKFPSYTATYQF) was added to the C-terminus of the construct via the EGGE linker. The EAAAK linker is considered a rigid linker for separating adjuvants from other parts of the vaccine sequence because of the special accessibility of the adjuvants and their interaction with TLRs. Furthermore, the GGGS, GPGP, KK, and EGGE linkers provide flexibility for fusing the epitopes [69]. These linkers were used to ensure the effective separation of individual epitopes and to inhibit the formation of junctional/neoepitopes and improve processing. Notably, the junctional epitopes hinder the efficacy of the selected epitopes [70]. The sequence of the vaccine model was screened through various servers, including VaxiJen, AllerTOP v.2.0, ToxinPred2 (Table 1g), TMHMM, and the peptide match server [59, 71].

### 2.10. Prediction of physicochemical and immunogenic properties

The ProtParam tool was used to analyze a variety of physicochemical properties. These properties include the number of amino acids, molecular weight, theoretical isoelectric point (pI), and estimated half-life (both in vitro and in vivo), as determined by the N-end rule, the aliphatic index, and the GRAVY index of the vaccine construct [30, 31, 72].

### 2.11. Secondary structure prediction

#### 2.11.1. SOPMA

The secondary structures of *Gag* were predicted using the online server SOPMA. All of the parameters used in SOPMA were configured to their default values. These default values include setting the number of conformational states to four, namely, helix, sheet, turn, and coil. The similarity threshold was set to eight, and the window width was set to 17 [31].

#### 2.11.2. RNAfold

Another instrument employed for the assessment of the secondary configuration of the DNA vaccine structures was RNAfold. The RNAfold server offers the most fundamental and widely utilized function. To utilize this service, the user must enter a single sequence in DNA/RNA format into a designated text field on the input form. In the simplest scenario, the server employs the classic Zuker and Stiegler algorithm [73] to predict only the minimum free energy (MFE) structure of the given sequence. Additionally, the server has the capability to calculate equilibrium base pairing probabilities using John McCaskill’s partition function algorithm [74]. By default, the RNA energy parameters from the Turner group [75] were utilized. The output of the fold server consists of a static HTML page that presents the predicted MFE structure as a string in brackets. It also includes links to plots that are generated for visualization purposes. There are three types of plots that can be produced. The predicted MFE structure is depicted as a conventional secondary structure graph using the naview layout method [76].

### 2.12. 3D modeling, refinement, and validation of the vaccine construct

To simulate the tertiary structures of the vaccine constructs, the I-TASSER server was used to produce five model sequences [55]. Subsequently, the optimized crude 3D models of the vaccine sequence were subsequently submitted to the GalaxyWEB server to reconstruct unreliable termini or loops of the initial model structures, thereby generating five refined models [40]. These models were established based on diverse parameters including GDT-HA, RMSD, MolProbity, Clash score, Poor rotamers, and Rama favored, serving as the foundation for template selection, sequence alignment, model construction, and enhancement. Consequently, additional online tools namely ERRAT [77], ProSA-Web [78], and RAMPAGE [79] were employed to identify the optimal model in this investigation. The ERRAT tool evaluates the general quality factor by considering the number of nonbonded interactions between various atomic types within a specific distance of 0.35 nm, with models scoring above 85 deemed favorable. Conversely, the RAMPAGE tool employs a Ramachandran plot to evaluate the stereochemical quality of the protein structure, aiming for a higher number of residues in the favored region and lower residues in the disallowed region. Furthermore, the Z score provided by the ProSA-Web server serves as an indicator of the overall quality of the model, where a positive Z score highlights potential structural issues or errors [24, 31].

### 2.13. Prediction of discontinuous BCL epitopes

A considerable number of BCL epitopes are not known to form a continuous sequence in the amino acid composition of a protein. These noncontinuous BCL epitopes assume a crucial function in biological operations [80]. ElliPro employs a trinity of algorithms to forecast disjunct epitopes. The program approximates the shape of the protein as an ellipsoid, computes the residue protrusion index (PI), and clusters the adjacent residues according to their PI values. ElliPro subsequently generates a PI score for every anticipated epitope [81]. In this particular investigation, the ElliPro server was used to forecast the noncontinuous BCL epitopes with a screening threshold of 0.5 for the vaccine [82].

### 2.14. Molecular docking

The process of presenting peptides derived from pathogens to T cells is strongly dependent on MHC molecules [83]. To evaluate the molecular docking process and the interaction performance between the expected CTL and HTL epitopes and their corresponding binding alleles, AutoDockVina software was used. To achieve this objective, we utilized the protein database (PDB) website to extract the PDB files of the most common alleles (MHC molecules) (refer to S1 and S2 Tables for more details). To determine the three-dimensional structure of these epitopes as ligands, we used the PEP-FOLD 4.0 server [84, 85].

### 2.15. Protein‒protein docking between TLR 2, 3, 4, 7, and 9 and the vaccine construct

It is of utmost importance to establish a stable correlation between a prospective vaccine and an immune receptor to initiate efficacious immune reactions. TLRs play a critical role in the initial protection against pathogens and serve as pivotal connectors between innate and adaptive immunity [83]. To achieve the desired immune response, it is crucial to evaluate the ability of the vaccine construct to attach to the immune receptor. Molecular docking, a computational technique, can be utilized to predict the binding affinity between the *Gag* vaccine construct and immune receptors, as well as the creation of interaction complexes [85]. Therefore, we performed molecular docking of the *Gag* vaccine construct to TLRs (TLR-2, TLR-3, TLR-4, TLR-7, and TLR-9) using the online server ClusPro 2.0. The PDB files for TLR-2 (PDB ID: 2Z7X), TLR-3 (PDB ID: 1ZIW), TLR-4 (PDB ID: 3FXI), TLR-7 (PDB ID: 5GMF), and TLR-9 (PDB ID: 3 WPB) were obtained from the RCSB database [24].

### 2.16. Molecular dynamic simulation

The use of MD simulation is widely recognized as a powerful method for studying biological systems at the molecular level [86]. In this study, we employed Linux-based GROMACS software to investigate the efficacy of the designed *Gag* vaccine against the TLR-2, TLR-3, TLR-4, TLR-7, and TLR-9 systems. The GROMACS tool can be implemented with a diverse range of force fields, including AMBER, OPLS, GROMOS, and CHARMM. For our research, the simulation was conducted using the GROMACS 2018—x series (version 05), employing the OPLS-AA force field (Optimized Potential for Liquid Simulation). During the preliminary stage of preparation, the *Gag* vaccine-TLR complexes were subjected to the integration of OPLS-AA force field parameters. As a result of this procedure, coordinate and topology files for the complex system are produced. The system was then solvated using the transferable intermolecular potential 3P (TIP3P) water model, followed by neutralization with Cl ions to ensure that the structural and topological coordinates remained stable [83]. Then, a process of energy minimization was performed, resulting in the final structure obtained through energy minimization (EM) [31] results were visualized using Excel software. The NVT ensemble equilibration lasted 100 ps and required 50,000 steps to reach the desired temperature. This process enabled the generation of velocities, allowing the simulation to run at different speeds. A 50,000-step NPT ensemble was then used to examine the density, potential, pressure, and temperature of the stabilized Gag vaccine construct throughout the process. After equilibration, a 100 ns, 50,000,000 step MD simulation was performed on the construct. The root mean square deviation (RMSD) of the backbone energy is minimized after MD simulation, and the results are presented in the form of a graph. Additionally, the radius of gyration (Rg), density plots, hydrogen bonding, and performance of the root mean square fluctuation (RMSF) protocol during MD simulations were analyzed.

### 2.17. Immune stimulation

We employed the C-IMMSIM online tool to analyze the immune stimulation effect of the vaccine on *HIV-1*. The strength of the interaction between a specific peptide-HLA complex and a T-cell receptor was assessed using the Miyazawa-Jernigan residue-residue potential tool [87]. C-IMMSIM was employed with its default settings [31], except for host HLA selection. The chosen HLA types were HLA-A*2402, HLA-A*3001, HLA-B*0701, HLA-B*4001, HLA-DRB1*1101, and HLA-DRB1*0101, as they have higher genotypic frequencies in the global population according to the IEDB analysis server.

### 2.18. Codon optimization and in silico cloning

**Fig 1:**
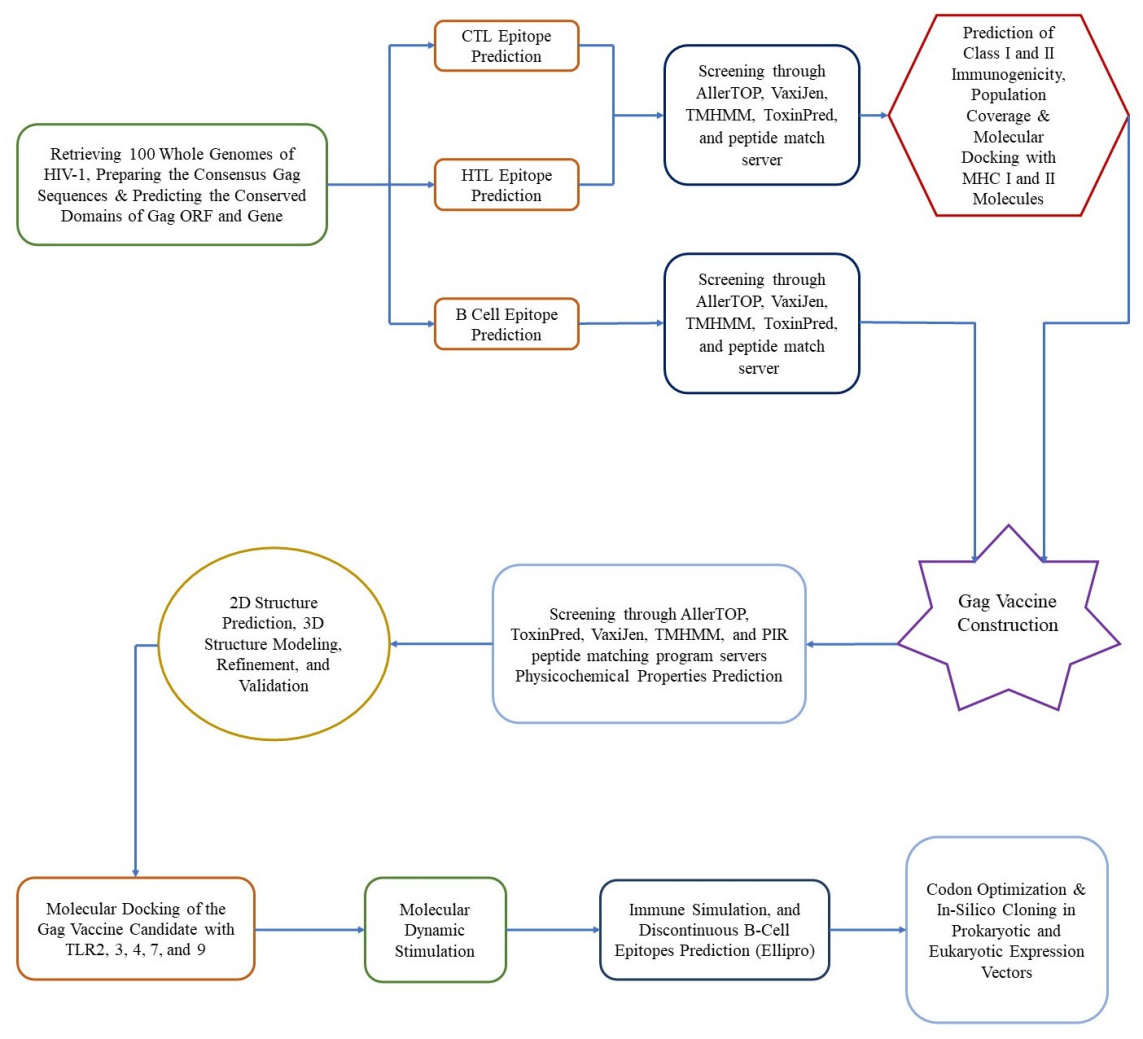
Flowchart of Gag vaccine design in this research.

Both the human and mouse immune systems were compatible with the epitopes of the vaccine model; therefore, eukaryotic and prokaryotic expression vectors were designed. A Kozak sequence that controls translation initiation and contains a start codon was added to the N-terminus of the vaccine protein of the eukaryotic expression vector vaccine. To increase the expression of the protein, codon optimization was performed for the *Homo sapiens* expression system using the VectorBuilder server (Table 1k). The cleavage sites of the *BamHI* and *EcoRV* restriction enzymes were excluded from the optimized cDNA sequence. Using SnapGene v7.1.1., the highly efficient reverse-transcribed nucleotide sequence, with a suitable GC content and CAI, was inserted into the pcDNA3.1(+) vector between the *BamHI* and *EcoRV* restriction sites under the control of the *CMV* promoter. Similarly, the prokaryotic vaccine construct was cloned in silico into the pET-30a (+) plasmid using the *EcoRI* and *EcoRV* restriction enzymes and codon optimization in the K12 strain of *E*. *coli*. In comparison to the pET-28a (+) plasmid, the pET-30a (+) plasmid contained a greater number of restriction sites, thereby conferring a distinct advantage in facilitating the expression of the target protein. The flowchart of the *Gag* vaccine design is shown in Fig 1.

The immunoinformatic techniques used in the development of the eukaryotic and prokaryotic in silico vaccines were the same, except for some techniques mentioned in the S3 Table.

## 3. Results

### 3.1. Protein sequence retrieval and alignment

A map of the distribution of the *HIV* subtypes and CRFs identified in the LANL database and the accession numbers of the full-length *HIV* sequence subtypes and CRFs used in the present study are shown in S1 Fig and S4 Table, respectively. The consensus sequence of *Gag* obtained one hundred main subtypes and CRFs was MGARASVLSGGKLDRWEKIRLRPGGKKKYRLKHIVWASRELERFAVNPGLLETSEGCRQILGQLQPALQTGSEELKSLYNTVATLYCVHQRIDVKDTKEALDKIEEEQNKSKKKAQQAAADTGNSSQVSQNYPIVQNLQGQMVHQAISPRTLNAWVKVIEEKAFSPEVIPMFSALSEGATPQDLNTMLNTVGGHQAAMQMLKETINEEAAEWDRLHPVHAGPIAPGQMREPRGSDIAGTTSTLQEQIGWMTSNPPIPVGEIYKRWIILGLNKIVRMYSPVSILDIRQGPKEPFRDYVDRFYKTLRAEQATQEVKNWMTETLLVQNANPDCKTILKALGPGATLEEMMTACQGVGGPGHKARVLAEAMSQVTNSNTIMMQRGNFRNQRKTVKCFNCGKEGHIARNCRAPRKKGCWKCGKEGHQMKDCTERQANFLGKIWPSHKGRPGNFLQSRPEPTAPPESFRFGEETTTPSQKQEPIDKELYPLASLKSLFGNDPSSQ.

### 3.2. Identification of conserved domains

The results showed that the HIV-1 Gag polyproteins contained five conserved domains comprising Gag_p17, Gag_p24_C, Gag_p6, the PTZ00368 superfamily, and Gag_p24, which are parts of the *HIV* nucleocapsid (Table 2). Epitopes located in the *Gag* conserved domains and passed through all the filters were qualified candidates for consideration in the vaccine construct.

**Table 2:** A list of conserved domains of the Gag gene sequence of HIV-1.

Genes Name	Conserved Domain(s)	Description	Interval
**Gag ORF**	Gag_p17	Gag gene protein p17 (matrix protein): The matrix protein forms an icosahedral shell associated with the inner membrane of the mature immunodeficiency virus.	2–132
	Gag_p24_C	Gag protein p24 C-terminal domain; p24 forms the inner protein layer of the nucleocapsid. ELISA tests for p24 are the most commonly used method to demonstrate virus replication both in vivo and in vitro.	278–349
	Gag_p6	Gag protein p6; HIV protein p6 contains two late-budding domains (L domains), which are short sequence motifs essential for viral particle release. p6 interacts with the endosomal sorting complex and represents a docking site for several cellular and binding factors. The PTAP motif interacts with the cellular budding factor TSG101. This domain is also found in some chimpanzee immunodeficiency virus (SIV-cpz) proteins.	449–485
	PTZ00368 superfamily	Universal minicircle sequence binding protein (UMSBP); Provisional	349–429
	Gag_p24	Gag gene protein p24 (core nucleocapsid protein); p24 forms the inner protein layer of the nucleocapsid. ELISA tests for p24 are the most commonly used method to demonstrate virus replication both in vivo and in vitro.	143–268

### 3.3. Identification of the biophysical and biochemical features of HIV genes

The ExPASy server’s ProtParam tool successfully predicted distinct physicochemical attributes of the Gag protein. S5 Table shows the findings of various physicochemical parameters pertaining to the Gag protein.

### 3.4. Linear BCL epitope prediction

**Fig 2:**
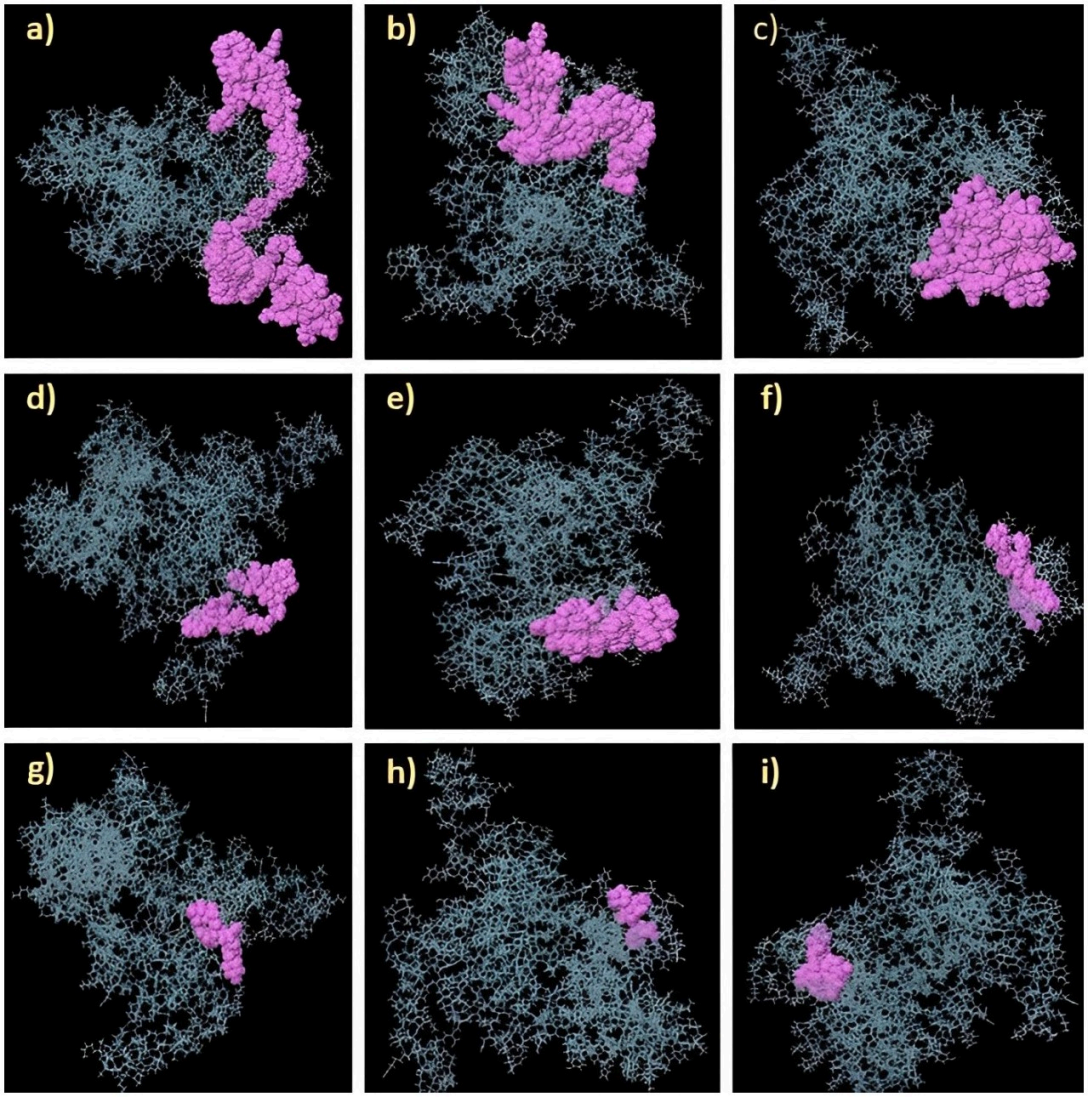
Linear B-cell epitopes predicted on the Gag vaccine construct (1–9). The purple color displays each linear B-cell epitope, such as amino acids 8–116, with a score ranging from 0.50–0.78.

Multiple methods were employed to identify the most proficient linear BCL epitopes for the ORF Gag protein. Among the plethora of suggested epitopes, only a finite number possessing desirable attributes, including antigenicity, no signal sequence, nontoxicity, nonallergen, and nonhomology to human proteins, were meticulously chosen (see Table 3, S6 Table, and Fig 2).

**Table 3:** A list of BCL-selected epitopes of the Gag vaccine.

Epitope	Software	Start	End	VaxiJen Score	Allergenicity	Toxicity	Topology (With or without signal sequence)	Homology to human proteins
LSGGKLDRWEKIRLRP	ABCpred	8	23	0.5113	Non allergen	Nontoxin	Without	Non-Homologous
RWEKIRLRPGGKKKYR	ABCpred	15	30	0.9114	Non allergen	Nontoxin	Without	Non-Homologous
GQLQPALQTGSEELKS	ABCpred	62	77	0.7717	Non allergen	Nontoxin	Without	Non-Homologous
QAAADTGNSSQVSQNY	ABCpred	117	132	0.4525	Non allergen	Nontoxin	Without	Non-Homologous
EEAAEWDRLHPVHAGP	ABCpred	207	22	1.1953	Non allergen	Nontoxin	Without	Non-Homologous
FRFGEETTTPSQKQEP	ABCpred	462	477	0.9054	Non allergen	Nontoxin	Without	Non-Homologous
TTPSQKQEPIDKELYP	ABCpred	469	484	0.7145	Non allergen	Nontoxin	Without	Non-Homologous

### 3.5. HTL epitope prediction

Epitopes that were suggested by both the IEDB MHC II and HLA binding webservers and possessed the capability to adhere to at least one mouse H-2-I allele and one human allele were subjected to a series of filters. These epitopes, which can bind to both human and mouse alleles concurrently, have the potential to elicit immune responses in both mice and humans. In the end, only those epitopes that were antigenic, lacked a signal sequence, were nonallergenic, nontoxic, nonhomologous and devoid of signal sequences that could induce IFN-γ responses were considered in the vaccine model (Table 4 and S1 Table).

**Table 4:** Potential antigenic and immunogenic HTL epitopes of the Gag vaccine.

Start	End	Epitope	Topology (With or Without signal sequence)	VaxiJen Score	Allergenicity	Toxicity	IFN-γ	Homology to human proteins
18	32	KIRLRPGGKKKYRLK	Without	1.4181	Non-Allergen	Non-Toxin	+	Non-Homologous
19	33	IRLRPGGKKKYRLKH	Without	1.3964	Non-Allergen	Non-Toxin	+	Non-Homologous
20	34	RLRPGGKKKYRLKHI	Without	1.2682	Non-Allergen	Non-Toxin	+	Non-Homologous
21	35	LRPGGKKKYRLKHIV	Without	0.8888	Non-Allergen	Non-Toxin	+	Non-Homologous
25	39	GKKKYRLKHIVWASR	Without	1.6425	Non-Allergen	Non-Toxin	+	Non-Homologous
86	100	YCVHQRIDVKDTKEA	Without	1.2952	Non-Allergen	Non-Toxin	+	Non-Homologous
165	179	SPEVIPMFSALSEGA	Without	0.5309	Non-Allergen	Non-Toxin	+	Non-Homologous

### 3.6. CTL epitope prediction

The NetCTL 1.2 server puts a large number of epitopes for the Gag protein targeting 12 MHC supertypes in the human population. The restricted epitopes were shown to be immunogenic, antigenic, nontoxic, nonallergenic, and nonhomologous to the human proteome and possessed suitable topology. Furthermore, epitopes situated in conserved domains were taken into account in the final formulation of the vaccine (Table 5 and S2 Table).

**Table 5:** Potential antigenic and immunogenic CTL epitopes.

Start	End	Epitope	Topology (With or Without signal sequence)	VaxiJen score	Allergenicity	Toxicity	Homology to human proteins
5	13	ASVLSGGKL	Without	0.4112	Non allergen	Non toxin	Non-Homologous
7	15	VLSGGKLDR	Without	0.7872	Non allergen	Non toxin	Non-Homologous
19	27	IRLRPGGKK	Without	1.0968	Non allergen	Non toxin	Non-Homologous
20	28	RLRPGGKKK	Without	1.1327	Non allergen	Non toxin	Non-Homologous
27	35	KKYRLKHIV	Without	0.4846	Non allergen	Non toxin	Non-Homologous
28	36	KYRLKHIVW	Without	1.6840	Non allergen	Non toxin	Non-Homologous
31	39	LKHIVWASR	Without	1.7391	Non allergen	Non toxin	Non-Homologous
43	51	RFAVNPGLL	Without	0.8171	Non allergen	Non toxin	Non-Homologous
83	91	ATLYCVHQR	Without	0.9807	Non allergen	Non toxin	Non-Homologous
84	92	TLYCVHQRI	Without	0.6599	Non allergen	Non toxin	Non-Homologous
124	132	NSSQVSQNY	Without	0.5278	Non allergen	Non toxin	Non-Homologous
144	152	HQAISPRTL	Without	0.7389	Non allergen	Non toxin	Non-Homologous
176	184	SEGATPQDL	Without	0.4833	Non allergen	Non toxin	Non-Homologous
179	187	ATPQDLNTM	Without	0.5071	Non allergen	Non toxin	Non-Homologous
210	218	AEWDRLHPV	Without	1.1628	Non allergen	Non toxin	Non-Homologous
253	261	NPPIPVGEI	Without	0.8100	Non allergen	Non toxin	Non-Homologous
308	316	QATQEVKNW	Without	0.4330	Non allergen	Non toxin	Non-Homologous
326	334	ANPDCKTIL	Without	0.5935	Non allergen	Non toxin	Non-Homologous

### 3.7. Population coverage

**Fig 3:**
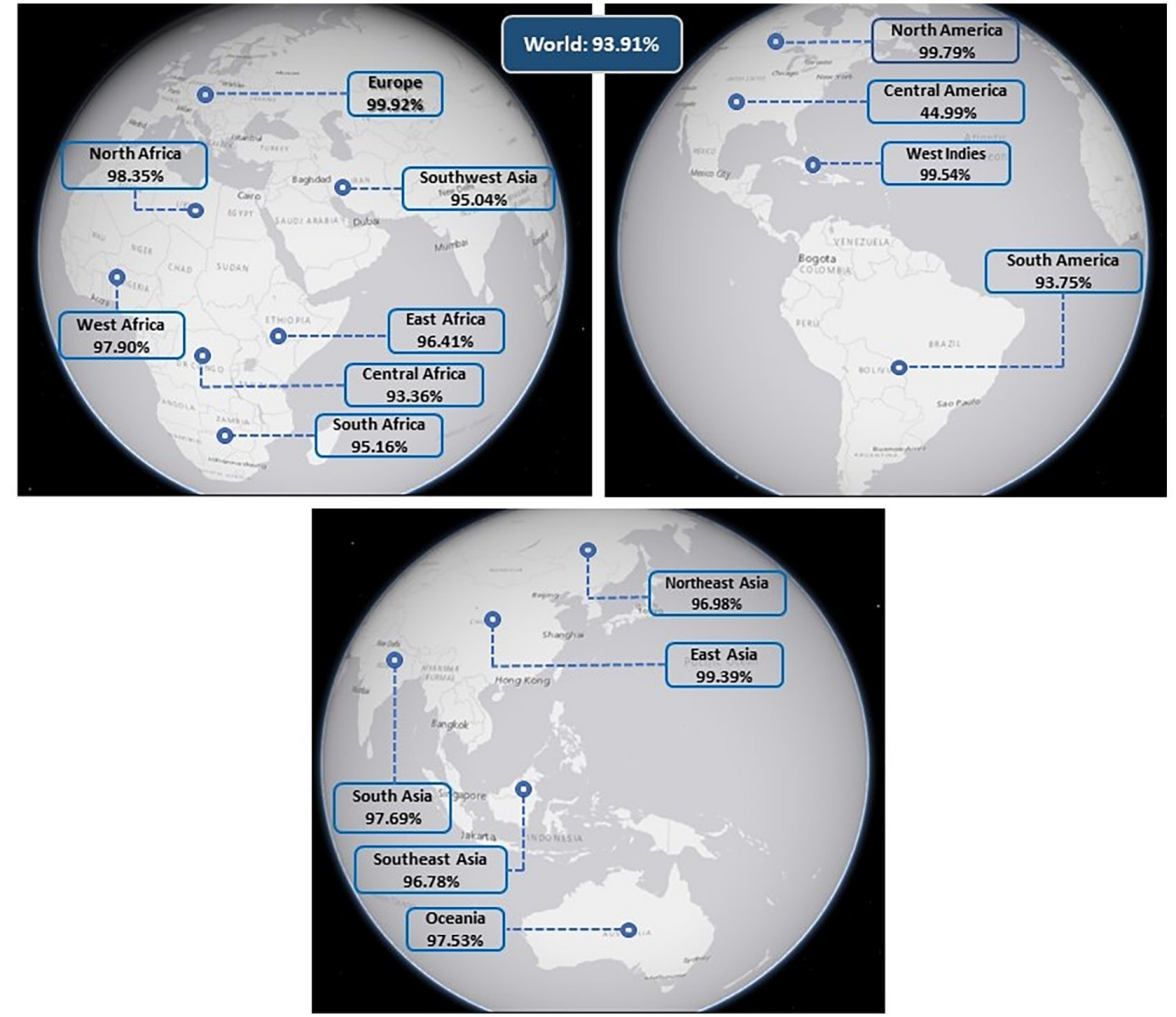
Percentage of combined (MHC I and MHC II) coverage of the selected epitopes (CTL and HTL) of the Gag vaccine in the global population (Generated by https://certmapper.cr.usgs.gov/data/apps/world-energy/?resource=continuous).

We used the IEDB population coverage tool to analyze the population coverage of 18 CTL epitopes and 7 HTL epitopes and their corresponding HLA alleles in 16 different regions around the world. The European nations exhibited the most elevated occurrence of the MHCI and II alleles, encompassing 99.92% of the *Gag* gene. On the other hand, combined alleles found in Central American countries were the least widespread (44.99%) among the *Gag* genes. Furthermore, when studying the impact of these epitopes on the population, 93.91% of the global population was revealed to be covered when considering both MHC class I and II. Fig 3, Table 1o, and S7 Table provide a visual representation of these data. The results regarding population coverage indicate that *HIV-1 Gag* vaccine candidates can combat the worldwide occurrence of *HIV* infection.

### 3.8. Vaccine construction

**Fig 4:**
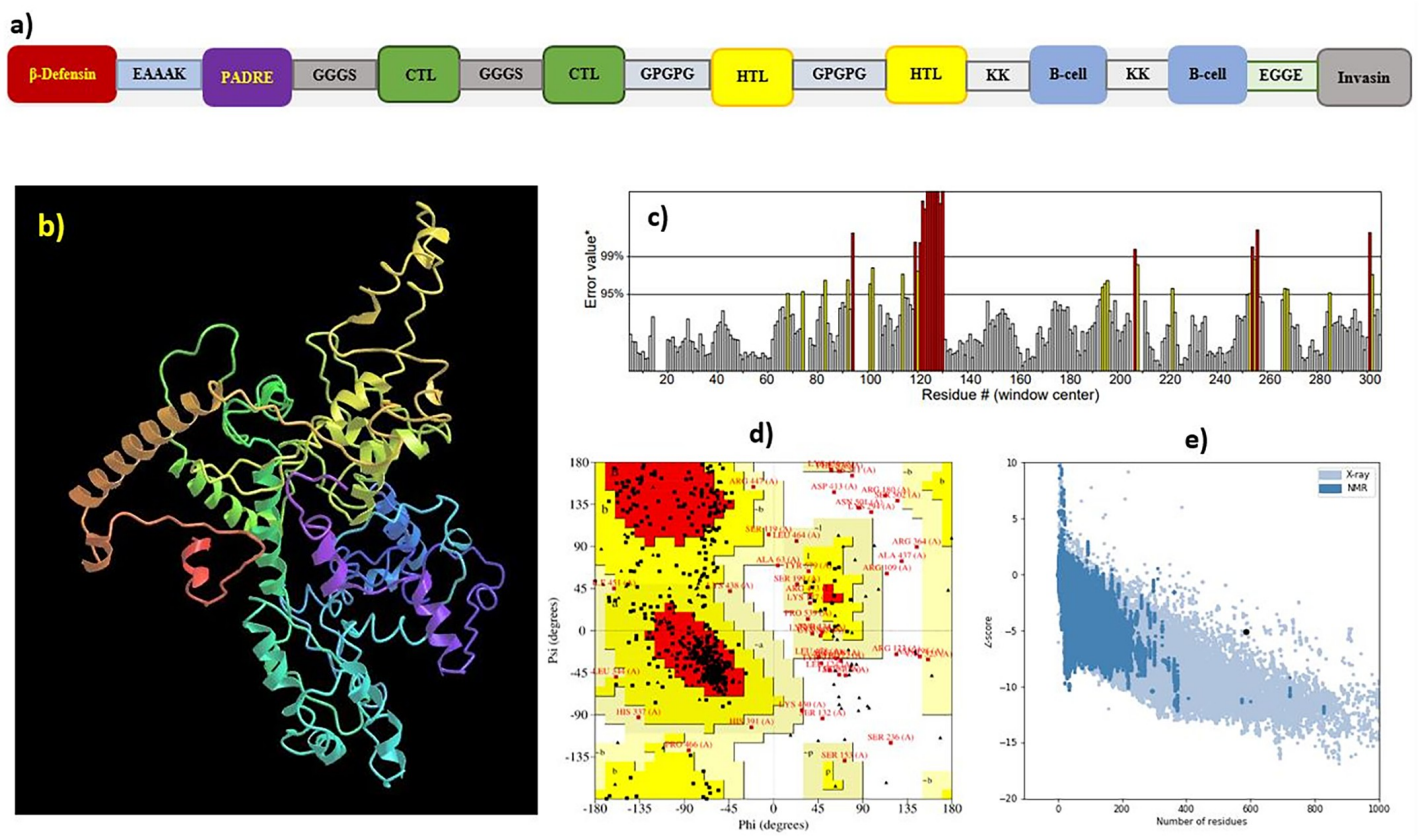
Gag vaccine designed: a) Proposed Gag-HIV vaccine construct; b) Best 3D model of designed Gag vaccine; c) Confirmation of vaccine structure defined by crystallography using the ERRAT tool; d) Ramachandran plot of the refined Gag vaccine was created with the PDBsum software; e) Protein structure analysis of vaccine designed by Prosa-web tool.

As illustrated in Fig 4a, a vaccine sequence consisting of multiple epitopes was designed by combining seven HTL epitopes, eighteen CTL epitopes, and seven BCLs (S8 Table). To ensure the efficient presentation of epitopes and maximum immunity in the body, each HTL, BCL, and CTL epitope was separated from the others via the GPGPG, KK, and GGGS linkers, respectively.

Beta defensin-3, a TLR4 agonist, was added at the beginning of the construct sequence that was attached to the PADRE sequence by the EAAAK linker, but this sequence was separated from CTL epitopes by the GGGS linker to guarantee maximum MHC-II allele coverage and induction of the CTL response. Finally, the C-terminal invasin sequence of Yersinia was incorporated into the vaccine model, to which the BCL epitopes were linked using an EGGE linker. Based on various servers, the vaccine sequence (GGSVLSGGKLDRGGGSIRLRPGGKKGGGSRLRPGGKKKGGGSKKYRLKHIVGGGSKYRLKHIVWGGGSLKHIVWASRGGGSRFAVNPGLLGGGSATLYCVHQRGGGSTLYCVHQRIGGGSNSSQVSQNYGGGSHQAISPRTLGGGSSEGATPQDLGGGSATPQDLNTMGGGSAEWDRLHPVGGGSNPPIPVGEIGGGSQATQEVKNWGGGSANPDCKTILGPGPGKIRLRPGGKKKYRLKGPGPGIRLRPGGKKKYRLKHGPGPGRLRPGGKKKYRLKHIGPGPGLRPGGKKKYRLKHIVGPGPGGKKKYRLKHIVWASRGPGPGYCVHQRIDVKDTKEAGPGPGSPEVIPMFSALSEGAKKLSGGKLDRWEKIRLRPKKRWEKIRLRPGGKKKYRKKGQLQPALQTGSEELKSKKQAAADTGNSSQVSQNYKKEEAAEWDRLHPVHAGPKKFRFGEETTTPSQKQEPKKTTPSQKQEPIDKELYPEGGETAKSKKFPSYTATYQF) appears to be safe for in vitro and in vivo studies. Moreover, the vaccine models were found to be nonhomologous to the human proteome; nontoxic, antigenic, and nonallergenic; and without any signal sequence (S9 and S10 Tables).

### 3.9. Secondary and tertiary structure prediction, refinement, and validation

#### 3.9.1. SOPMA

Table 1b shows a variety of values pertaining to the anticipated parameters crucial for determining the secondary structures of the Gag protein, all of which were obtained through the utilization of the SOPMA online server. The secondary structure of the aforementioned protein exhibited areas characterized by alpha helix (45.49%) formations, with the exception of the transactivator protein, as well as elongated strands (7.62%), beta turns (4.41%), and random coil (42.48%) conformations (S11 Table).

#### 3.9.2. RNAfold

The structure of the mRNA vaccine was deduced through the use of the RNAfold platform. Additionally, the computation of the structures’ free energy was performed using this specific website. As an initial step, the codons that were optimized for utilization in the vaccine were selected. The mRNA vaccine displayed a minimal free energy of -545.80 kcal/mol during production, while the energy of its secondary centroid structure was recorded as -384.01 kcal/mol. The outcomes suggest that the structure of the mRNA vaccine is expected to exhibit stability. The optimal secondary structure is shown in dot-bracket notation, with a minimum free energy of -545.80 kcal/mol. The free energy of the thermodynamic ensemble is -569.08 kcal/mol. The frequency of the MFE structure in the ensemble is 0.00%. The ensemble diversity is 464.70. centroid secondary structure is shown in dot-bracket notation with a minimum free energy of -384.01 kcal/mol.

### 3.10. Tertiary structure prediction, refinement, and validation

To generate the 3D model of the vaccine, the I-TASSER server employed comparative modeling techniques, resulting in the production of a total of 5 vaccine construct models. The reliability of each model was assessed using the C-score, which quantifies the precision of the suggested models. As a result, the model with the most favorable C-score was chosen for further refinement. The refinement process of the selected model involved the utilization of the GalaxyRefine server (Fig 4b–4e). Additionally, quality analysis of the ProSA web server demonstrated an improved Ramachandran plot, wherein the majority of residues were located in the most favored region (Fig 4d). Overall, the model exhibiting high-quality three-dimensional characteristics was deemed suitable because of the 3D structure of the vaccine.

### 3.11. Prediction of discontinuous B-bell epitopes

**Fig 5:**
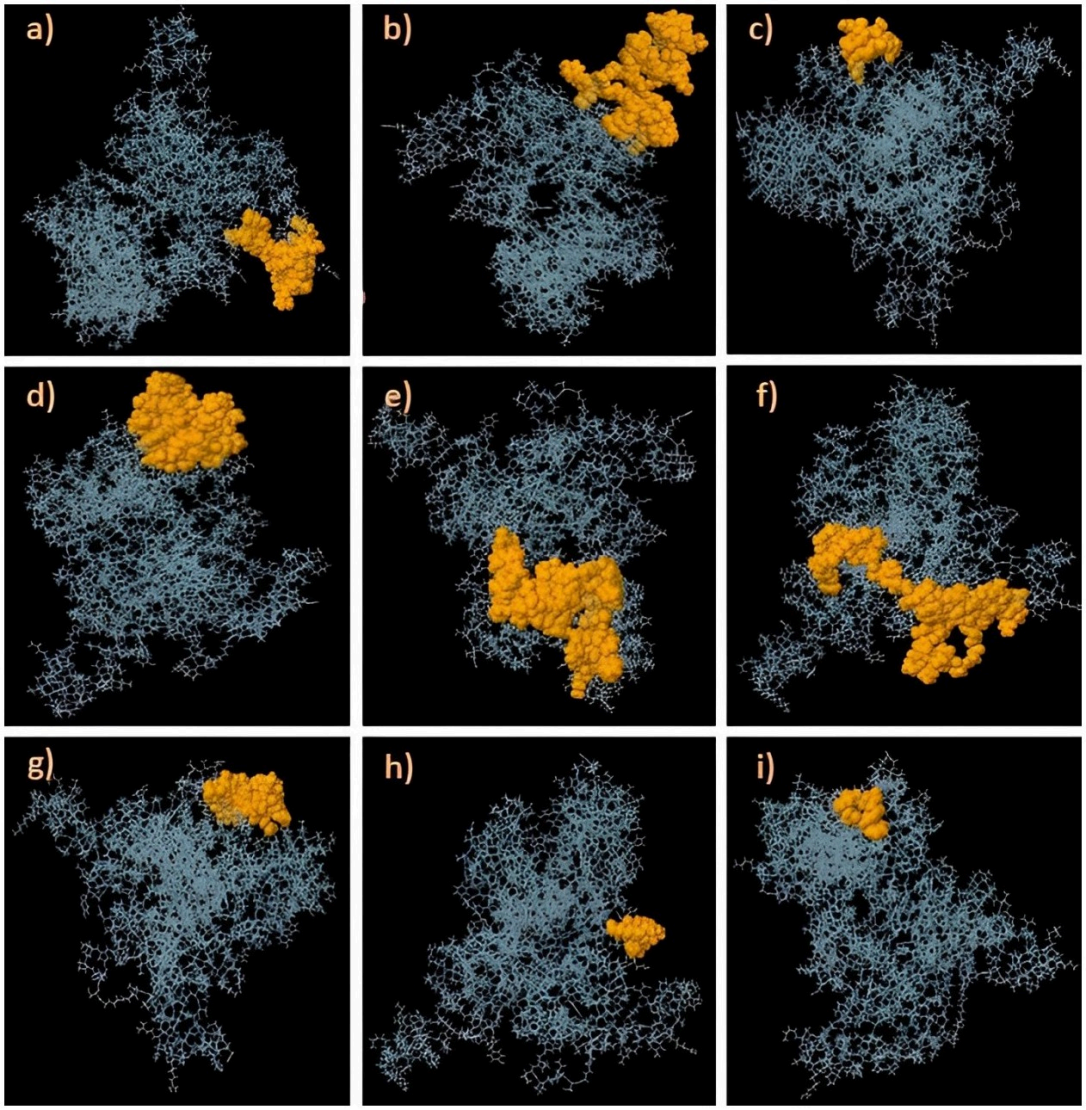
Discontinuous B-cell epitopes on the vaccine construct (1–6) are indicated by the orange region of the Gag vaccine, which depicts each discontinuous B-cell epitope comprising residues 4 to 63 with score values ranging from 0.532 to 0.841.

The existence of efficacious BCL epitopes plays a crucial role in the framework of vaccines, as it facilitates the elicitation of humoral immunity against exogenous pathogens [88]. The ElliPro tool, employed at the IEDB, successfully detected a single epitope within the vaccine construct. This epitope is characterized by a residual position ranging from 62 to 77 and has a score of 0.679. (Table 6, S12 Table and Fig 5).

**Table 6:** Discontinuous epitopes found in the Gag vaccine construct overlap with linear epitopes.

Overlapped Discontinuous Epitopes	Final Linear BCL epitopes	Start and end positions in vaccine construct
A:A62, A:A63, A:G64, A:G65, A:G66, A:S67, A:A68, A:S69, A:V70, A:L71, A:S72, A:G73, A:G74, A:K75, A:L76, A:G77	GQLQPALQTGSEELKS	62–77

### 3.12. Molecular docking of the T-cell epitopes with MHC molecules

We performed a molecular docking analysis in our study to investigate how individual T-cell epitopes interact with their corresponding alleles. The details of these interactions can be found in S1 and S2 Tables. The data in Table 7 demonstrate that the binding affinity between CTL epitopes and the corresponding alleles ranged from -8.6 to -6.1 kcal/mol. Conversely, the binding affinity range between the selected HTL epitopes and their alleles, as indicated by the AutoDock Vina docking results, was between -6.7 and -5.5 kcal/mol. The data revealed that the CTL epitope (NSSQVSQNY) and the HTL epitope (IRLRPGGKKKYRLKH) of the *Gag* gene exhibited the highest affinity for binding when paired with their corresponding MHC alleles (HLA-A*01:01 and HLA-DRB1*0101).

**Table 7:** AutoDock VINA results between the CTL and HTL epitopes of Gag and related alleles.

Epitopes		1	2	3	4	5	6	7	8	9	10	11	12	13	14	15	16	17	18
**Binding Energy**	**CTL+MHC I**	-6.9	-6.3	-8.5	-6.2	-6.7	-6.9	-8	-7.7	-6.5	-6.8	-8.6	-7.2	-6.7	-6.1	-7.1	-7.5	-8	-6.3
	**HTL+MHC II**	-6.5	-6.7	-6.3	-5.8	-5.6	-5.4	-5.4											

### 3.13. Protein‒protein docking between TLRs and vaccine constructs

**Fig 6:**
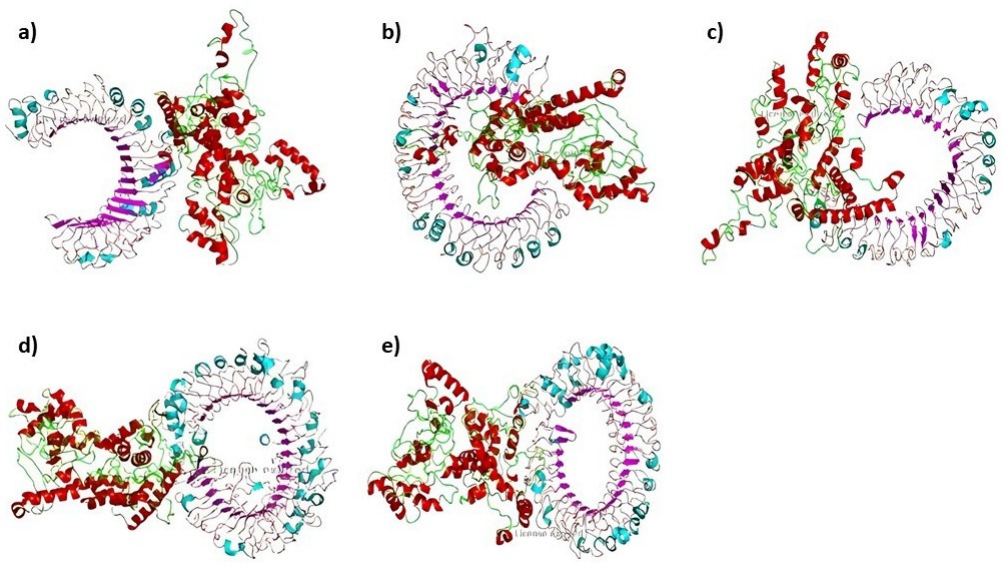
Docked complex of the Gag vaccine construct with a) TLR-2, b) TLR-3, c) TLR-4, d) TLR-7, and e) TLR-9. The 3D molecule of the vaccine is shown in green and red according to the ClusPro server.

ClusPro 2.0 was utilized to investigate the interaction between TLRs and the *Gag* vaccine structure via protein‒protein docking. A set of 30 models was generated for each docking, and the best energy scores were used to select the models that effectively engaged with the receptor. The docking results showed that the *Gag* vaccine construct had the lowest energy scores when interacting with TLR-2, TLR-3, TLR-4, TLR-7 or TLR-9, with recorded values of -1106.9, -1336.9, -1287.4, -1317.8, and -1272.7, respectively (as stated in S13 Table. The complex formation data obtained by docking indicated that the *Gag* vaccine construct, as designed, exhibited the highest affinity for binding with all five TLRs at the lowest magnitude (as shown in Fig 6a–6e).

### 3.14. Molecular dynamic analysis

**Fig 7:**
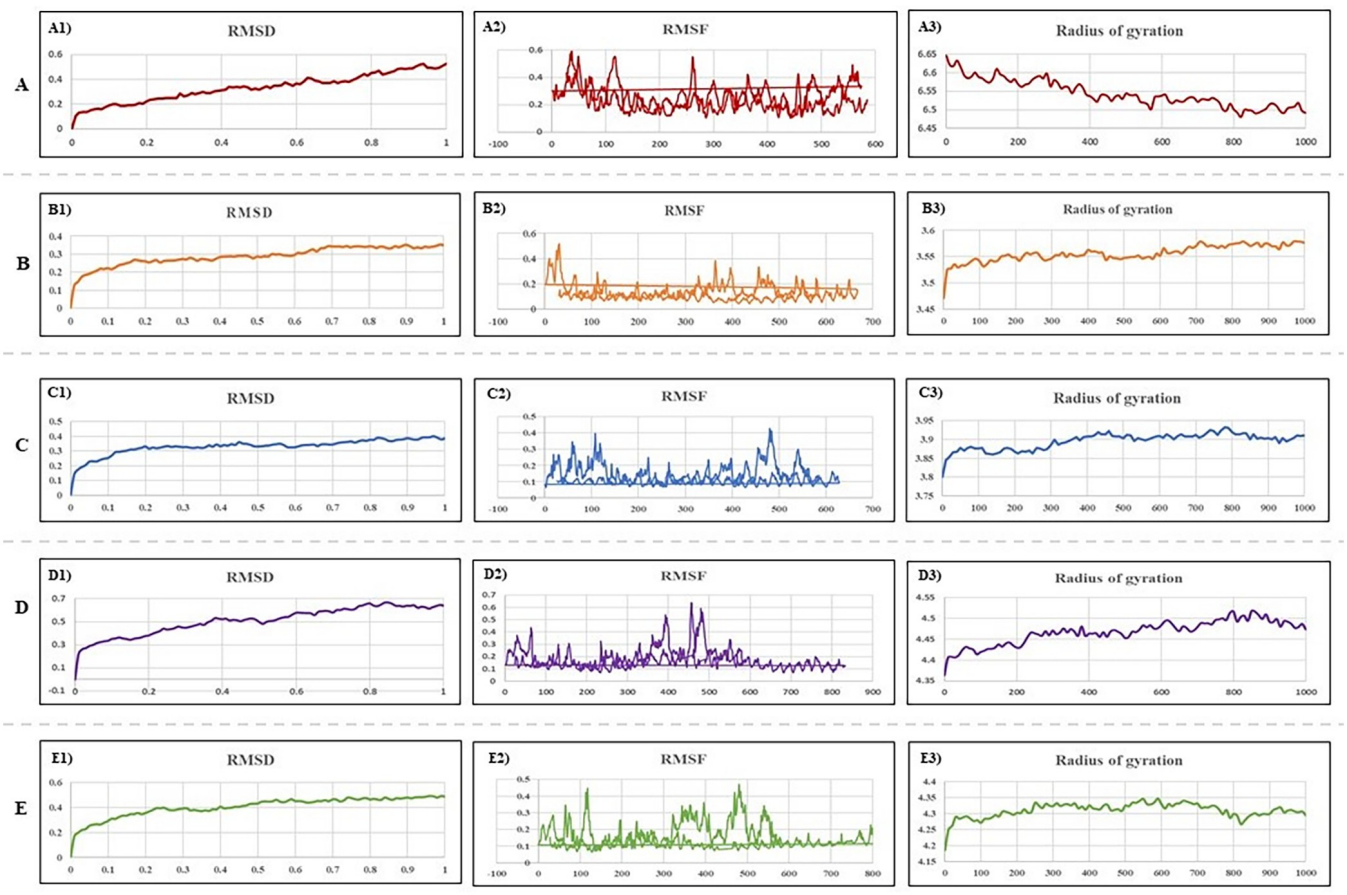
Molecular dynamics simulation analysis of A) TLR-2, B) TLR-3, C) TLR-4, D) TLR-7, and E) TLR-9 with Gag vaccine constructs. RMSD results (A1-E1); RMSF results (A2-E2); Rg results (A3-E3).

The results of molecular dynamics simulations of *Gag* vaccine docking complexes with TLR-2, TLR-3, TLR-4, TLR-7, and TLR-9 are shown in Fig 7(A)–7(E). It was predicted that the simulations could determine the movement of atoms and molecules within the vaccine structure. Furthermore, the *HIV-1/Gag* vaccine construct was evaluated via various calculations, including density assessment, energy minimization, pressure evaluation, potential energy determination, temperature analysis, and Rg computation. By examining the trajectory produced during a 100 ns simulation, we acquired the RMSD and RMSF values. The *Gag* vaccine, which was designed to include TLRs (specifically TLR-2, TLR-3, TLR-4, TLR-7, and TLR-9), demonstrated RMSD values of 0.95 nm, 0.91 nm, 0.97 nm, 0.98 nm, and 0.96 nm, respectively, indicating its stability. The average RMSD value for the vaccine during the simulation was 0.95 nm [Fig 7(A1)–7(E1)]. Furthermore, the average RMSF value for the complex of the vaccine construct and the TLRs was 0.47 nm, confirming the overall stability of the protein structure throughout the MD simulation [Fig 7(A2)–7(E2)]. Rg demonstrated the structural stability of the construct during the MD simulation [Fig 7(A3)–7(E3)]. This finding implies that the flexibility and stability of the vaccine-TLR complex are balanced, which is beneficial for immune receptor activity.

### 3.15. Immune stimulation

**Fig 8:**
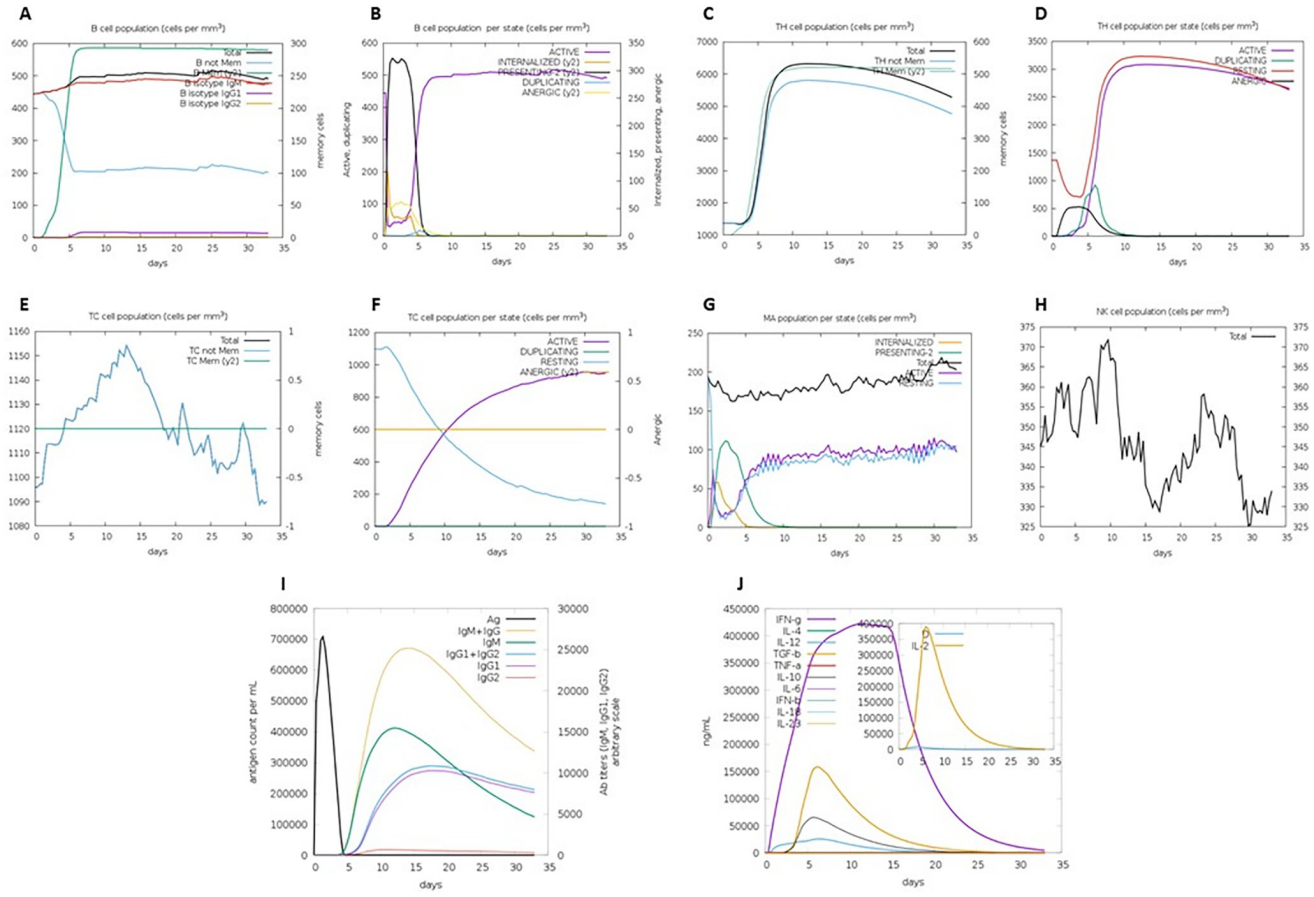
Immune simulation outcomes obtained from C-IMMSIM after administration of the Gag vaccine are shown in plots a-j.

Fig 8A–8J displays the outcomes of the immune simulation for the *Gag* vaccine design. Following the administration of the vaccine, various immune responses, including the production of IgM and IgG, the concentration of cytokines, the levels of BCL and Th-cell populations, and specifically, the generation of IgM+IgG antibody titers, were observed. Furthermore, the vaccine enhanced the population of IL-2 cells, memory cells, and Th cells. In addition, we noticed an increase in the quantity of dendritic cells, macrophages (MAs), and NK cells when the vaccine we created was used to stimulate the immune system. The immune simulation results showed that innate immunity and cytokine-based immune regulation are involved. MAs, a part of the innate immune system, are crucial for triggering an inflammatory reaction when exposed to antigens. As a result, their activation leads to the release of certain cytokines, such as IL-12 and IL-18, which in turn stimulate the adaptive immune response and the production of IFN-γ to promote immune activation. Consequently, these potential options can elicit effective immune responses.

### 3.16. In silico cloning of the vaccine candidates

**Fig 9:**
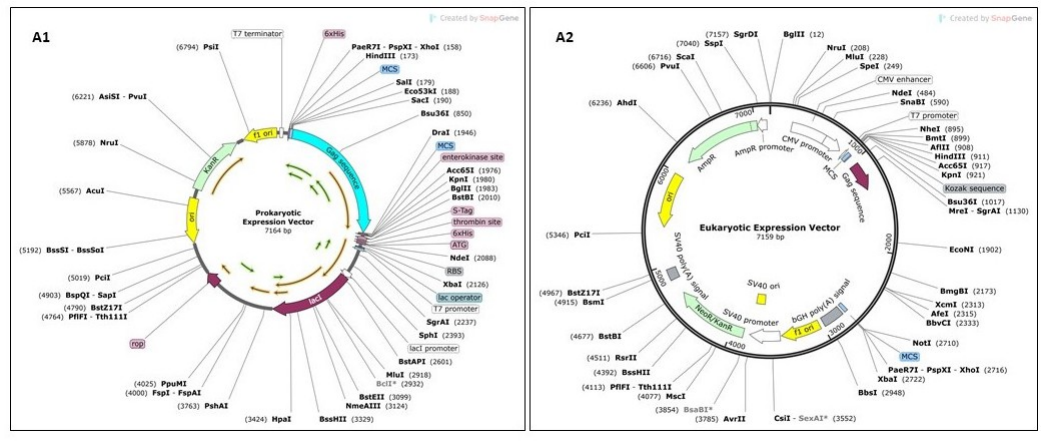
In silico cloning of the Gag vaccine construct A1) pET-30a(+) plasmid and A2) pcDNA3.1(+) vector.

The GC content and CAI of the optimized cDNA derived from humans and the K12 strain of *E*. *coli* were determined to be 59.99% and 0.89 and 59.99% and 0.92, respectively. The ideal value for the GC content is 30–70%, and the CAI is 1 [89]. The results demonstrated the successful and efficient expression of cDNA sequences from vaccine models in both eukaryotic and prokaryotic systems. The optimized sequences, identified in S14 Table, were subsequently inserted into the pcDNA3.1(+) vector and pET-30a(+) plasmid under the control of the *CMV* promoter using SnapGene v7.1.1 (Fig 9A1). To construct the pcDNA3.1(+) vector containing the *HIV-Gag* vaccine model, a *BamHI* restriction site was added at the 5’ end of the vaccine sequence, followed by the insertion of the Kozak sequence (Fig 9A2). A stop codon (TAA) was added at the 3’ end of the optimized codon, along with an *EcoRV* restriction site. To prepare pET-30a(+), which contains the sequence of the *HIV* vaccine construct. *EcoRI* and *EcoRV* restriction sites were inserted into the N- and C-termini of the optimized vaccine sequences, respectively.

## 4. Discussion

Given the increasing incidence and spread of emerging and re-emerging infections, the low effectiveness and high cost of many drugs, scientists are exploring new methods, namely, vaccination, to provide a cost-effective and effective cure or eradicate pathogens [90]. In addition, immunization against pathogens protects individuals from acquiring the disease and facing complications, resulting in wellness and enhanced physical conditions in human [91–93]. Recently, bioinformatic tools have been extensively employed in analyzing medical databases, leading to a considerable generation of experimental data intended for the development of novel vaccines [82]. Progress in the domains of reverse vaccinology, computational biology, and immunoinformatic presents an ideal opportunity to facilitate the design of vaccines that are not only safe and effective but also reduce the time and cost involved [94–96]. Furthermore, genomic and proteomic data are utilized to investigate potential epitopes for the development of long-lasting immunogenic subunit vaccines [97–99]. For example, several studies have focused on the development of multiepitope vaccines for different diseases caused by viruses such as bovine leukemia virus [100], dengue [101], Kaposi sarcoma [102], SARS-CoV-2 [103, 104], West Nile virus [86], and monkey poxvirus [83]. Therefore, a multiepitope vaccine that can effectively stimulate safe and strong immune responses is suggested for numerous infections [59, 105]. The complexity of comprehending the human immune response to the *HIV* pathogen poses a significant obstacle in the search for vaccines [85]. Pandey et al. applied innovative immunoinformatic methods in their research to develop a multi-epitope subunit vaccine for HIV. The vaccine includes B-cell and T-cell epitopes that can trigger both humoral and cell-mediated immune responses. It was determined that the vaccine is non-allergenic, safe, and has the potential to stimulate an immune response. The vaccine design exhibited strong interaction with TLR-3, an immune receptor that activates immune cells. In silico cloning was carried out to confirm the expression of the vaccine in microbial systems [44]. Habib et al. conducted a study in which they designed a vaccine for HIV-1 using bioinformatic tools, molecular docking, and MD simulations. The vaccine construct consisted of 315 amino acids and had the potential for HIV-1 prevention. Different prediction tools were utilized to examine the physiochemical properties and secondary structure of the vaccine, suggesting stability and possible immunogenicity. The researchers also looked into proteasomal cleavage and transporter-associated antigen processing to improve epitope specificity and sensitivity in the design of the vaccine [106]. The ineffectiveness of *HIV* vaccines can be attributed to their inability to induce helper T-cell and cellular responses, as demonstrated in trials of the in silico vaccine EP HIV-1090 [25]. Additionally, these vaccines have proven to be ineffective against rapidly mutating *HIV* infection [44]. Trials on BALB/c mice have shown that multiepitope vaccines are incapable of producing broadly neutralizing antibodies [107]. *HIV* vaccines fail to activate the desired innate immune response or induce appropriate cytokines. Furthermore, the effectiveness of the *HIV* vaccine is constrained by its capacity to stimulate the immune system in response to a few *HIV* genotypes. In a study carried out by Pandey et al., the designed vaccine-induced immune responses only against specific subtypes of *HIV*, namely, C and B [44]. Moreover, *HIV-1* has a reputation for substantial genetic heterogeneity, thereby resulting in notable dissimilarities among the protein sequences of different *HIV* subtypes and CRFs. The effectiveness of vaccine constructs can be improved globally by considering the sequences of the major *HIV* subtypes and CRFs [108]. To address this issue, for the first time, new in silico vaccine models against the full length of the *HIV-Gag* genome were developed that were compatible with the main *HIV* subtypes and CRFS.

The designed vaccine constructs present promising avenues to address the specific challenges faced in controlling HIV infection including: 1) Selection of qualified epitopes from Gag conserved domains to target immunodominant responses to overcome the rapid mutation rate of HIV and enhance the ability to inhibit multiple subtypes and CRFs of HIV-1 by stimulation of the broadly neutralizing antibodies and cellular responses. 2) Induction of stronger cellular, humoral, and innate immune responses compared to vaccines without beta defensin-3 which is as TLR3 adjuvant an adjuvant. 3) Amplification of the immune response against HIV by incorporating several sequences, including beta defensin-3, universal PADRE, and C-terminal invasin sequence of Yersinia. 4) Selection of epitopes that were immunogenic, antigenic, nontoxic, nonallergenic, and nonhomologous to the human proteome and possessed suitable topology that were joint together with elements such as linkers for optimal epitope presentation. 5) Designing a new vaccine model ensuring broader global population coverage based on a consensus sequence of various HIV subtypes and CRFs.

This study was performed with the aim of developing a secure vaccine suitable for both human and mouse hosts. In particular, the research team obtained a collection of one hundred complete *HIV* gene sequences belonging to the major subtypes and CRFs from the LANL. Using immunoinformatic methods, we identified potential CTL, HTL, and BCL epitopes within the full-length Gag sequence. Given the pivotal role that Gag plays in the intricate life cycle of *HIV-1*, *Gag* is the logical focus of vaccine development [109]. For example, the *HIV*-Gag protein has various functions in the replication and life cycle. It participates in the formation of the viral capsid, transportation to the plasma membrane, interaction with host factors, and packaging of the viral genome [110, 111]. In this regard, a consensus *Gag* sequence utilized for vaccine design was derived from 100 *Gag* sequences representing the most prevalent subtypes and CRFs. In the context of designing epitope-based vaccines, the utilization of highly conserved epitopes can confer a wider range of immune protection against various subtypes, CRFs and even different strains. This notion is supported by previous research [85]. Hence, the final epitopes were chosen based on conserved regions within the Gag protein to generate a more robust and comprehensive immune response against the major subtypes and CRFs. A protein domain is characterized as the autonomously evolved, operative, and structurally preserved segment of a protein sequence that serves as a functional and structural representation of the corresponding protein. Consequently, the conserved domain may serve as an innovative target for the development of vaccines [85].The epitopes that were examined in this investigation have the capacity to generate neutralizing antibodies targeting the *HIV-Gag* gene. Furthermore, they also have the ability to elicit a suitable immune response involving the production of IFN-ɣ and activation of the innate immune system by interacting with the Toll-like receptors TLR-2, 3, 4, 7, and 9.

BCL epitopes play a significant role in the development of an immune response capable of combating viral infections. These epitopes possess distinct characteristics that enable BCLs to detect and trigger immune responses specific to viral infections [76, 112]. In this study, five different methods were utilized to identify linear BCL epitopes, followed by a screening process. The ElliPro server was also employed to predict both the conformation and linear BCL epitopes of the vaccine construct. Taken together, these findings indicate that the vaccine model has the capacity to induce humoral immunity and effectively identify *HIV* infection.

Protection against *HIV* is closely linked to T cells, which can stimulate various cytokines [113]. The presentation of antigens to CTLs through MHC-I/HLA-I is a critical step in triggering the immune response and generating memory cells to combat diseases. These CTLs are essential in the MHC-I-mediated immune response because they are responsible for identifying and eliminating damaged, virus-infected, or cancerous cells through the epitopes presented by the MHC-I/HLAI molecules on the cell surface. For this purpose, MHC-I/HLAI epitopes must possess significant immunogenicity to activate CD8+ T lymphocytes [114]. The results of these studies indicate that T cells that can stimulate the production of various cytokines have a significant impact on the suppression of *HIV* infection and could be vital in the development of efficacious vaccines [115].

Here, *Gag* was targeted by CTLs to find immunogenic CD8+ T-cell epitopes. The selected epitopes that can bind to both mouse and human alleles were passed through various filters and ultimately included in the vaccine construct. Detailed information can be found in S2 and S9 Tables. Another objective of this report was to evaluate potential HTL epitopes that can stimulate the production of IFN-ɣ and thereby initiate strong HTL immune responses through vaccination. The outcome of infectious diseases and the effectiveness of vaccines are influenced by various factors, including the induction of CD4+ cells, which can affect the virologic, host physiological, or molecular level [93, 112, 114, 116–119]. HTLs can acquire Th1 or Th2 phenotypes and activate immune responses, thereby stimulating macrophages, natural killer cells, and CD8+ T cells [99, 120, 121]. Moreover, HTLs are responsible for inducing robust humoral and cellular responses by facilitating the optimal expansion and maintenance of CD8+ T cells [122]. Therefore, *HIV-1* clinical trials have revealed that CD4+ T-cell populations are suitable candidates for use in vaccine models, as such cells can stimulate strong immune responses against *HIV* infection [123]. In this study, highly qualified HTL epitopes were identified through advanced computational biology techniques.

In this report, we screened all the primary epitopes using various filters. To evaluate the antigenicity of each epitope and vaccine construct, VaxiJen v2.0 was used to determine the antigens that induce the immune system in nature. Another factor evaluated was immunogenicity, which refers to antigens’ ability to stimulate the immune system without binding to T cells. The antigenic and immunogenic features of vaccine constructs and epitopes are essential in designing efficient vaccines [124]. ToxinPred and ToxinPred2 servers (Table 1g) were applied to assess the toxicity of the peptide and vaccine construct, respectively. According to our data, the finalized epitopes and the sequence of the vaccine construct were nontoxic.

Another quality factor that was screened was non-allergenicity. A safe human vaccine model should not induce an allergenic reaction resulting in skin rash, sneezing, wheezing, or swelling of the mucous membrane [57]. Finally, the presence of signal peptides and transmembrane helices within the epitopes was estimated using the DeepTMHMM server to exclude epitopes that contained either signal peptides or transmembrane helices [125]. To increase the efficacy of the vaccine constructs, specific features, such as the Kozak sequence found exclusively in eukaryote expression vectors and the beta-defensin, PADRE, and invasin sequences, were incorporated into both prokaryotic and eukaryotic expression vectors.

From the standpoint of a public health program, the effectiveness of a vaccine depends on various factors, including its specificity and extent of population coverage [126]. The analysis of population coverage verified that utilizing the last 25 epitopes led to 93.91% coverage of the global population. These findings suggest that creating a vaccine with multiple epitopes could offer enhanced protection against *HIV-1* infection. Notably, the MHC class combined epitopes exhibited the highest coverage in Europe (99.92%) and North America (99.79%). Conversely, Central America had the lowest coverage, with only 44.99% of the population. The reason for the lower population affected by HIV-1 in Central America compared to other parts of the world is mainly due to the prevalence of subtype B in the region, which makes up 98.9% of the sequences [127]. This high level of consistency indicates that HIV-1 subtype B was likely introduced to Central America only once, resulting in a more contained epidemic with separate outbreaks in each country [128]. On the other hand, regions like Latin America have a variety of HIV-1 subtypes and recombinants, which can affect coverage rates due to the complexity of the virus [129, 130]. Furthermore, the lack of visibility of the HIV epidemic among indigenous populations in Latin America also adds to the difficulty in achieving broader population coverage [131]. Together, these factors underscore the distinctive genetic diversity and epidemiological dynamics of HIV-1 in Central America, contributing to the lower coverage rates compared to more diverse regions globally. As a result, the constructed Gag vaccine provides widespread coverage for a vast number of people worldwide.

After all the required criteria were met, 32 epitopes were attached together using a variety of linkers in the vaccine construct. The utilization of the EAAAK linker in the designed vaccine ensures the separation of epitopes within the host [57, 70]. Moreover, the adjuvant was attached to the multiepitope vaccine through the EAAAK linker to minimize potential interactions among functional domains of the vaccine model [132]. To separate the CTL epitopes from one another, as well as from the PADRE sequence, a GGGS linker was used. The KK linker was utilized to bind BCL epitopes, ensuring the preservation of independent immunogenic responses [133]. The linkers GGGS, GPGPG, K, EAAAK, and EGGE have been extensively utilized in various bioinformatic methods for the development of multiepitope vaccines for viruses [94, 99]. Accordingly, the abovementioned linkers were used to join different sequences of the vaccine candidates.

In this study, adjuvants, as immunological and pharmacological substances, were added to the vaccine to stimulate and strengthen the adaptive immune system [134]. Defensins mediate a number of immune responses, including cell maturation, which generally leads to innate immunity [135]. In particular, defensins recruit naïve T cells and immature dendritic cells to specific sites of infection via the CCR6 receptor to trigger an adaptive immune response and exert anti-*HIV-1* activities and immunomodulatory effects [136]. Another adjuvant attached to the construct was PADRE, which stimulates the innate and humoral immune systems. It also reaches a larger population since such a peptide overcomes barriers, as indicated by the high diversity among HLA molecules [135]. According to previous studies, PADRE improves the presentation of epitopes that generate specific CTLs as vaccines for hepatitis B virus infection. Furthermore, it enhances the immune response to human papillomavirus vaccines by inducing a robust CD8+ response [137, 138]. To ensure the stable expression of vaccine constructs in human cells, the Kozak sequence upstream of optimized cDNA should be incorporated for the recognition of mRNA by ribosomes [139]. In the present report, the Kozak sequence was included in the vaccine model to increase the expression of the vaccine protein in human cells. In addition, the desirable properties of the designed vaccine were determined based on its physicochemical parameters.

Furthermore, the findings obtained from the secondary and tertiary structures of the vaccine model demonstrated a high level of immunogenicity. This result signifies the potential of the designed vaccine construct as a promising candidate for the development of an efficacious vaccine. In addition, the physicochemical parameters of the vaccine model illustrated the desirable properties of the construct.

The validation parameters for evaluating the accuracy of the 3D structure of the designed vaccine construct revealed that the improved 3D model of the *HIV* vaccine construct exhibited a favorable level of quality. Notably, the interaction between the T-cell epitopes and MHC molecules was examined through molecular docking. The docking findings indicated that the T-cell epitopes could effectively attach to MHC molecules, resulting in recognition by antigen-presenting cells. Molecular docking analysis revealed that the designed *HIV-1 Gag* vaccine exhibited strong affinity for TLRs (TLR-2, TLR-3, TLR-4, TLR-7, and TLR-9). These findings confirm that the immune system of humans and mice can be used to efficiently identify multiepitope vaccines, which can lead to consistent and potent immune responses. Previous investigations have established that TLRs assume a vital function in the beginning of innate immune responses due to their possession of the capacity to recognize pathogens and stimulate the evolution of the adaptive immune system [140]. For example, TLR-2 and TLR-4 identifies viral structural proteins and induces the generation of inflammatory cytokines, while TLR-3 stimulates the initiation of dendritic cell activation mediated by *HIV-1* [85]. Unexpectedly, the time factor plays a significant role in the response of TLR7 to *HIV*. During the initial phase of *HIV* infection, the activation of TLR7 leads to increased production of type I IFNs and a decrease in the expression of *HIV* coreceptors CCR5 and CXCR4 in CD4+ T cells [141]. This implies that stimulation of TLR7 inhibits the production of HIV and enhances the antiviral response of activated T cells, macrophages, and monocytes by inducing the production of type I IFNs [141, 142]. On the other hand, TLR3 has the ability to detect both double-stranded RNA (dsRNA) and single-stranded RNA (ssRNA) [143, 144]. Additionally, TLRs are capable of binding to polyproteins and epitopes [145–147]. For instance, TLR3 functions as a receptor for developing a vaccine that can target and identify the virus [94, 148]. The vaccine contains a TLR3 agonist known as β-defensin, which is connected to the N-terminus with an EAAAK linker [100]. TLR4 can activate immune responses against viruses by identifying proteins on the virus surface [59]. According to earlier studies, *HIV* can activate certain receptors (TLR-2, -3, -4, -7, and -9), which in turn activate downstream pathways that lead to the generation of proinflammatory cytokines. These cytokines play a role in fighting against *HIV* infection [24].

In this research, both prokaryotic and eukaryotic hosts were chosen for the production of the vaccine protein. Codon optimization was another step in this study. Each amino acid can be represented by multiple codons due to codon degeneracy, and the selection of synonymous codon pairs varies across different species. In other words, particular organisms or species tend to favor certain specific synonymous codons known as optimal codons [149]. As a result, codon optimization was conducted in both hosts to increase the efficiency of transcription and translation. This was achieved through an analysis of the total GC content as well as the codon adaptation index of the DNA sequence [150].

The stability of the vaccine-TLR complex was verified through MD simulation analysis, which included diverse environmental conditions, including changes in pressure and temperature. The RMSD analysis of the docked complexes revealed minimal deviation, indicating a stable interaction between the vaccine construct and TLR molecules. The RMSF graphs for the docked protein complexes displayed consistent levels, showcasing the flexibility of the side chains. Finally, the Rg plots for the docked complexes exhibited a steady curve, indicating stable vaccine-receptor complexes. Hence, the initial trajectory assessment, which involved calculating Rg, RMSD, RMSF, and hydrogen bonds, all supported the exceptional stability of the vaccine-TLR complexes in a biological context. Moreover, compared to those of receptor-vaccine complexes, the vaccine TLR4 construct displayed the highest level of stability when exposed to natural conditions. Using an immune simulator server, we predicted how the immune system would respond to three injections of the candidate Gag vaccine. Our analysis of the cytokine simulation plot showed an increase in IFN-γ, IL-2, TGFβ, IL-10, and IL-12 levels, similar to what Sher et al. [31] observed in the design of an *HIV-1* multiepitope vaccine (Fig 8J). This indicates that antigen-presenting cells were appropriately activated, leading to a high production of memory cells from BCLs and T cells. The cytokines produced by Th memory cells also help control and clear antigens. Furthermore, the persistence of long-term memory after three injections confirmed the effectiveness of our candidate vaccine.

Here, there are more explanation how the selected epitopes from the Gag polyproteins contribute to the overall efficacy of the vaccine. The efficacy of vaccines is significantly influenced by the selection and presentation of epitopes that are recognized by the immune system. The computational approaches towards vaccine design against Gag-HIV gene commenced with identifying highly antigenic, immunogenic, and non-allergenic epitopes specific to CTLs, HTLs, and linear and conventional B lymphocytes derived from virus whole Gag protein sequences. This step underscores the critical role these epitopes play in eliciting a robust immune response by targeting different arms of the adaptive immune system—CTLs for cellular immunity, HTLs for assisting other cells in the immune response including B cells for antibody production, and B lymphocytes directly responsible for antibody production. Following epitope selection, linkers were employed to connect these epitopes cohesively while adjuvants were introduced at the construct to enhance immunogenicity further. This strategy illustrates how selected epitopes contribute not only through their inherent properties but also through strategic assembly enhancing overall vaccine effectiveness. Moreover, molecular docking and dynamics simulations revealed strong and stable binding interactions between these vaccine candidates and human TLRs. The interactions are pivotal since TLRs play a crucial role in innate immunity as one of their primary sensors triggering downstream signaling pathways leading to inflammatory responses—a foundational step towards adaptive immunity activation where epitope-specific responses are generated. Additionally, computer-aided immune simulation predicted real-life-like immune responses upon administration, highlighting how these carefully chosen epitopes could potentially translate into effective real-world outcomes. Finally, yet importantly, codon optimization was performed on these vaccine candidates, facilitating their cloning into both prokaryotic and eukaryotic vectors for future experimental validation.

To the best of our knowledge, there has been no research on the specific targeting of Gag polyproteins to determine the BCL, CTL, and HLA epitopes to develop an in-silico vaccine. The study available, conducted by Manalu et al. in 2023 [151], focused on a small part of the Gag polyproteins. In this research, various immunoinformatic techniques were used to determine whether the p17 matrix protein could serve as a target for designing an in-silico vaccine against *HIV* infection. Christy et al utilized immunoinformatic methods to find only the CD4+ epitopes present in the Gag protein restricted to the HLA-DRB1*07 allele, which is widely prevalent among the Indian population [152]. Multiple experimental studies have been conducted to examine various proteins encoded by the Gag polyprotein sequence of *HIV* and simian immunodeficiency viruses (*SIVs*). In fact, *SIV*s are diverse viruses that naturally infect a variety of African primates, making them valuable animal models for studying the natural steps of *HIV* infection and disease [153]. Benlahrech et al. explored two approaches to assess the effectiveness of the CD8+ T-cell response using SIV *Gag* vaccine models [153, 154]. In 2023, Tarres-Freixas et al. reported that immunogenicity is enhanced by *Gag*-based virus-like particles (VLPs) compared with the soluble Gag protein [155].

The evaluation of bioinformatic vaccine efficacy is an intricate process that involves multiple stages, including antigen selection, immune response prediction, vaccine formulation design, and assessment through both computational simulations followed by rigorous experimental validations both in vitro/in vivo settings before proceeding towards clinical trials where actual effectiveness can be gauged among populations at risk [156]. For example, algorithms have been developed to predict T-cell epitopes’ ability to bind to MHC molecules and B-cell epitope recognition patterns. These predictions are important to designing a vaccine that elicits strong cellular immunity and humoral responses [157]. Additionally, computer models are used to simulate the dynamics between different parts of vaccines, such as adjuvants, with the immune system. This helps improve effectiveness while maintaining safety standards [158].

Planned experimental studies on HIV bioinformatic vaccines should underscore a multidisciplinary approach combining virology, immunology, bioinformatics, and biostatistics to improve our understanding of HIV’s interaction with the human immune system. Here, some suggestions can be considered: 1. Development of safe and effective HIV vaccines by evaluating multiple vaccine approaches in vivo that elicit cross-reactive humoral and cellular responses 2. Induction of specific cellular and humoral immune responses through vaccination without adverse effects and ensuring broad protection across all major global HIV strains. 3. Utilization of advanced statistical models in evaluating longitudinal changes post-vaccination. 4. Conduction of clinical trials studying in both adult and infant populations. In other words, the final goal lies within human trials, where real-world efficacy is assessed against circulating HIV subtypes and CRFs in diverse epidemiological settings to address the HIV pandemic.

The main focus of this research was to identify epitope-based vaccines that can trigger the infiltration of various immune cells and stimulate chemokine production in both humans and mice, as proven through the use of a molecular simulation technique. Diverse factors of the vaccine construct, including antigenicity, stability, thermostability, non-allergenicity, and hydrophilicity, were evaluated via immunoinformatic approaches. Immune stimulation has been shown to promote the development of memory cells via the activation of dendritic cells and macrophages in a vaccine model. Overall, the proposed vaccine models revealed great potential as vaccine candidates for controlling *HIV* infection. Three levels of research can be conducted, related to the present report: 1) The suggested vaccine models should be developed in a wet laboratory, 2) the efficacy of vaccine models should be checked on different *HIV* virus subtypes and CRFs, and 3) further investigations of vaccine efficacy can be designed and run-in clinical trials.

## 5. Conclusions

Therapeutic measures aimed at preventing and eradicating *HIV-1* infection necessitate innovative pharmaceuticals and immunization vaccines that can protect individuals at risk of pathogens and those already infected.

For the first time, in silico vaccine models against the *HIV-Gag* polyprotein against major *HIV* subtypes and CRFs were designed. A vast number of epitopes were predicted and screened using different parameters, and the qualified epitopes located in the conserved domains of the *Gag* sequence were selected for joining via appropriate linkers in vaccine models. The designed vaccines also contained suitable adjuvants to vigorously stimulate the immune response and reduce HLA polymorphisms in the population. The confirmation of the high level of expression of the vaccine models in both human cells and *E*. *coli* bacteria was achieved through the implementation of codon optimization. Furthermore, the vaccine constructs exhibited favorable affinity for the immune receptor TLR-2, TLR-3, TLR-4, TLR-7, and TLR-9. The final constructs were able to stimulate immune cells vigorously, which was confirmed using the immune stimulation method. Experimental analysis should be carried out in a wet laboratory setting to ascertain the effectiveness of the designed vaccine models.

## Supporting information

S1 Table A list of HTL-selected epitopes and identified MHC alleles in the Gag gene of HIV-1. (DOCX)

S2 Table A list of CTL-selected epitopes and identified MHC alleles in the Gag gene of HIV-1. (DOCX)

S3 Table The techniques that were different in the development of vaccines in eukaryotic and prokaryotic hosts. (DOCX)

S4 Table Accession numbers of 100 full-length HIV-1 sequences of the most predominant subtypes and CRFs. (DOCX)

S5 Table ProtParam results for the HIV Gag protein and vaccine construct. (DOCX)

S6 Table The residue data and scores of the predicted linear BCL epitopes on the Gag vaccine construct. (DOCX)

S7 Table Population coverage results of selected CTL and HTL epitopes in the Gag vaccine construct. (DOCX)

S8 Table Numbers of predicted CTL, HTL, and BCL epitopes. (DOCX)

S9 Table Results of screening the Gag vaccine construct. The sequence of the vaccine model was antigenic, nontoxic, nonallergenic, and nonhomologous to the human proteome and lacked any signal sequence.
(DOCX)

S10 Table The consensus sequences of the HIV-1 Gag gene and vaccine construct. (DOCX)

S11 Table Various features of the secondary structures of the HIV Gag gene and vaccine construct. (DOCX)

S12 Table The residue data and scores of the predicted discontinuous BCL epitopes on the Gag vaccine construct. (DOCX)

S13 Table Molecular docking results of TLRs and the Gag vaccine construct generated using the Cluspro2.0 tool. (The best models are marked with a green box).
(DOCX)

S14 Table The sequence of the optimized codon of the vaccine models in human and *E*. *coli* hosts. (DOCX)

S1 Fig Worldwide report of submitted subtypes and CRF sequences of HIV-1. The figure was adjusted from the LANL database.
(TIF)
