# Supplementary material for: Design of multivalent-epitope vaccine models directed toward the world’s population against HIV-Gag polyprotein: Reverse vaccinology and immunoinformatics
Source: PLoS One. 2024 Sep 27;19(9):e0306559. doi: 10.1371/journal.pone.0306559 (PMC11432917; doi:10.1371/journal.pone.0306559)
Supplement: S1 Table — (DOCX) [file pone.0306559.s001.docx]

**Table S1.** A list of HTL-selected epitopes and identified MHC alleles in the Gag gene of HIV-1

| **No.** | **Mouse Allele** | **Epitope** | **Start** | **End** | **MHC-II Allele (IEDB Percentile Rank ≤ 10)** | **PDB-ID** |
| --- | --- | --- | --- | --- | --- | --- |
| **1** | H2-IEd | KIRLRPGGKKKYRLK | 18 | 32 | HLA-DRB1*13:02, HLA-DRB1*03:01, HLA-DRB1*12:01, **HLA-DRB1*11:01** | **6CPL*** |
| **2** | H2-IEd | IRLRPGGKKKYRLKH | 19 | 33 | HLA-DRB1*13:02, HLA-DRB1*03:01, **HLA-DRB1*01:01** | **1AQD** |
| **3** | H2-IEk | RLRPGGKKKYRLKHI | 20 | 34 | HLA-DRB1*13:02, **HLA-DRB1*01:01** | **1AQD** |
| **4** | H2-IEd | LRPGGKKKYRLKHIV | 21 | 35 | HLA-DRB1*13:02, **HLA-DRB1*01:01** | **1AQD** |
| **5** | H2-IEd | GKKKYRLKHIVWASR | 25 | 39 | HLA-DRB1*08:02, HLA-DPA1*01:03/DPB1*02:01, **HLA-DRB1*11:01**, HLA-DQA1*04:01/DQB1*04:02, HLA-DRB1*13:02, HLA-DPA1*02:01/DPB1*05:01, HLA-DPA1*03:01/DPB1*04:02, HLA-DPA1*01:03/DPB1*04:01, HLA-DRB3*02:02, HLA-DRB5*01:01, HLA-DPA1*02:01/DPB1*14:01, HLA-DRB1*04:01, HLA-DPA1*02:01/DPB1*01:01, HLA-DRB1*01:01 | **6CPL** |
| **6** | H2-IAd | YCVHQRIDVKDTKEA | 86 | 100 | HLA-DRB1*03:01, HLA-DRB1*08:02, **HLA-DRB1*01:01** | **1AQD** |
| **7** | H2-IEk | SPEVIPMFSALSEGA | 165 | 179 | **HLA-DRB1*04:01**, HLA-DRB1*01:01, HLA-DRB1*15:01, HLA-DRB1*04:05, HLA-DRB1*08:02, HLA-DRB1*11:01, HLA-DRB4*01:01, HLA-DRB1*12:01, HLA-DRB5*01:01, HLA-DRB3*02:02, HLA-DQA1*04:01/DQB1*04:02, HLA-DQA1*01:01/DQB1*05:01, HLA-DQA1*03:01/DQB1*03:02, HLA-DPA1*03:01/DPB1*04:02 | **5NI9** |
| *** The bold font identified the Human HLA and the corresponding ID used in docking between epitopes and alleles.** | | | | | | |
