# Supplementary material for: Design of multivalent-epitope vaccine models directed toward the world’s population against HIV-Gag polyprotein: Reverse vaccinology and immunoinformatics
Source: PLoS One. 2024 Sep 27;19(9):e0306559. doi: 10.1371/journal.pone.0306559 (PMC11432917; doi:10.1371/journal.pone.0306559)
Supplement: S2 Table — (DOCX) [file pone.0306559.s002.docx]

**Table S2.** A list of CTL-selected epitopes and identified MHC alleles in the Gag gene of HIV-1

| **No.** | **Mouse Allele** | **Epitope** | **Start** | **End** | **MHC-I Allele (IEDB Percentile Rank ≤ 10)** | **PDB-ID** |
| --- | --- | --- | --- | --- | --- | --- |
| **1** | H-2-Qa1 | ASVLSGGKL | 5 | 13 | **HLA-B*07:02**, HLA-B*58:01, HLA-B*15:01, HLA-A*30:02, HLA-B*57:01, HLA-A*32:01, HLA-A*26:01, HLA-B*40:01, HLA-A*68:02, HLA-A*30:01, HLA-B*51:01, HLA-A*01:01, HLA-A*02:06, HLA-B*44:02, HLA-A*02:03, HLA-B*53:01, HLA-B*44:03, HLA-A*11:01, HLA-A*24:02, HLA-B*35:01, HLA-A*02:01, HLA-A*23:01, HLA-A*68:01, HLA-A*03:01 | **5EO0*** |
| **2** | H-2-Qa1 | VLSGGKLDR | 7 | 15 | HLA-A*31:01, **HLA-A*03:01,** HLA-A*33:01, HLA-A*11:01, HLA-A*68:01, HLA-A*30:01, HLA-A*32:01, HLA-A*30:02, HLA-A*02:01, HLA-A*01:01 | **7L1C** |
| **3** | H-2-Kb | IRLRPGGKK | 19 | 27 | **HLA-A*30:01,** HLA-A*03:01, HLA-A*11:01, HLA-A*30:02, HLA-A*31:01, HLA-A*68:01, HLA-A*33:01, HLA-B*44:02 | **6J1W** |
| **4** | H-2-Qa1 | RLRPGGKKK | 20 | 28 | **HLA-A*30:01**, HLA-A*03:01, HLA-A*31:01, HLA-A*11:01, HLA-A*30:02, HLA-A*32:01, HLA-B*15:01, HLA-B*07:02, HLA-A*33:01, HLA-A*68:01, HLA-A*02:03, HLA-B*44:02, HLA-B*57:01, HLA-A*02:01, HLA-B*44:03, HLA-A*01:01, HLA-A*26:01, HLA-B*58:01, HLA-B*40:01, HLA-A*02:06 | **6J1W** |
| **5** | H-2-Kb | KKYRLKHIV | 27 | 35 | **HLA-B*40:01,** HLA-A*30:01, HLA-B*44:03, HLA-B*08:01, HLA-A*32:01, HLA-B*44:02, HLA-B*51:01, HLA-A*30:02, HLA-B*07:02, HLA-A*23:01, HLA-B*58:01, HLA-A*24:02, HLA-B*15:01, HLA-A*02:06, HLA-A*68:02 | **6IEX** |
| **6** | H-2-Dd | KYRLKHIVW | 28 | 36 | **HLA-A*24:02**, HLA-A*23:01, HLA-A*32:01, HLA-B*57:01, HLA-B*58:01, HLA-B*08:01, HLA-A*30:01, HLA-A*30:02, HLA-A*31:01, HLA-B*53:01, HLA-B*44:02, HLA-B*07:02, HLA-A*33:01, HLA-B*44:03, HLA-B*15:01, HLA-A*03:01, HLA-A*26:01, HLA-B*35:01, HLA-B*40:01, HLA-B*51:01 | **7JYV** |
| **7** | H-2-Kd | LKHIVWASR | 31 | 39 | HLA-A*33:01, HLA-A*31:01**, HLA-A*30:01,** HLA-A*68:01, HLA-A*03:01, HLA-A*11:01 | **6J1W** |
| **8** | H-2-Kd | RFAVNPGLL | 43 | 51 | **HLA-A*24:02**, HLA-A*23:01, HLA-B*07:02, HLA-A*30:02, HLA-A*30:01, HLA-A*32:01, HLA-B*08:01, HLA-B*40:01, HLA-B*58:01, HLA-B*15:01, HLA-A*31:01, HLA-B*57:01, HLA-A*02:03, HLA-A*03:01, HLA-B*53:01, HLA-A*26:01, HLA-B*44:02, HLA-B*44:03, HLA-B*35:01, HLA-B*51:01 | **7JYV** |
| **9** | H-2-Qa1 | ATLYCVHQR | 83 | 91 | HLA-A*31:01, **HLA-A*11:01,** HLA-A*68:01, HLA-A*33:01, HLA-A*03:01, HLA-A*30:01, HLA-A*32:01, HLA-A*30:02, HLA-A*26:01, HLA-A*01:01, HLA-B*57:01, HLA-A*68:02, HLA-B*58:01, HLA-B*44:03, HLA-B*44:02, HLA-A*02:06, HLA-A*24:02, HLA-A*23:01 | **5WJN** |
| **10** | H-2-Kb | TLYCVHQRI | 84 | 92 | HLA-A*02:03, **HLA-A*02:01,** HLA-A*32:01, HLA-B*51:01, HLA-A*02:06, HLA-A*68:02, HLA-A*23:01, HLA-B*08:01, HLA-A*24:02, HLA-B*15:01, HLA-A*26:01, HLA-B*57:01, HLA-B*58:01, HLA-A*30:01, HLA-B*53:01, HLA-A*30:02, HLA-A*03:01, HLA-B*40:01, HLA-A*31:01, HLA-A*01:01, HLA-A*33:01, HLA-B*44:02, HLA-B*44:03, HLA-A*11:01 | **4U6Y** |
| **11** | H-2-Qa1 | NSSQVSQNY | 124 | 132 | **HLA-A*01:01**, HLA-A*30:02, HLA-B*35:01, HLA-A*26:01, HLA-B*53:01, HLA-B*15:01, HLA-B*58:01, HLA-B*57:01, HLA-A*68:01, HLA-B*44:03, HLA-B*44:02, HLA-A*32:01, HLA-A*11:01, HLA-A*68:02, HLA-A*03:01, HLA-B*51:01, HLA-A*33:01, HLA-B*40:01, HLA-A*30:01, HLA-A*31:01, HLA-A*24:02, HLA-A*23:01, HLA-B*07:02, HLA-B*08:01, HLA-A*02:06 | **6MPP** |
| **12** | H-2-Qa2 | HQAISPRTL | 144 | 152 | **HLA-B*15:01**, HLA-B*40:01, HLA-B*08:01, HLA-A*02:06, HLA-A*32:01, HLA-B*44:02, HLA-B*44:03, HLA-B*07:02, HLA-A*02:03, HLA-A*30:02, HLA-A*30:01, HLA-A*24:02, HLA-A*23:01, HLA-B*35:01, HLA-A*02:01, HLA-B*51:01, HLA-B*53:01, HLA-B*58:01, HLA-A*26:01, HLA-B*57:01, HLA-A*68:02, HLA-A*01:01, HLA-A*03:01, HLA-A*31:01, HLA-A*33:01, HLA-A*11:01, HLA-A*68:01 | **6UZP** |
| **13** | H-2-Qa2 | SEGATPQDL | 176 | 184 | **HLA-B*40:01**, HLA-B*44:02, HLA-B*44:03, HLA-A*24:02, HLA-B*07:02, HLA-B*35:01, HLA-B*08:01, HLA-B*15:01, HLA-A*26:01, HLA-B*53:01, HLA-A*23:01, HLA-A*02:06, HLA-B*51:01, HLA-A*01:01 | **6IEX** |
| **14** | H-2-Db | ATPQDLNTM | 179 | 187 | HLA-A*26:01, **HLA-A*02:06**, HLA-B*58:01, HLA-A*24:02, HLA-B*57:01, HLA-B*15:01, HLA-A*01:01, HLA-A*68:02, HLA-A*23:01, HLA-B*35:01, HLA-A*32:01, HLA-A*30:02, HLA-A*02:03, HLA-A*02:01, HLA-B*53:01, HLA-B*07:02, HLA-B*40:01, HLA-B*51:01, HLA-A*11:01, HLA-B*08:01, HLA-A*68:01, HLA-B*44:03, HLA-B*44:02, HLA-A*30:01, HLA-A*33:01, HLA-A*03:01, HLA-A*31:01 | **3OXR** |
| **15** | H-2-Qa2 | AEWDRLHPV | 210 | 218 | **HLA-B*40:01**, HLA-B*44:02, HLA-B*44:03, HLA-A*02:06, HLA-A*02:01, HLA-B*08:01, HLA-A*02:03, HLA-A*68:02, HLA-A*26:01, HLA-A*32:01, HLA-B*51:01, HLA-A*33:01, HLA-B*15:01, HLA-A*30:02, HLA-B*07:02, HLA-A*23:01, HLA-A*01:01, HLA-A*24:02, HLA-B*35:01, HLA-B*53:01, HLA-A*30:01, HLA-A*11:01, HLA-A*31:01, HLA-A*03:01, HLA-A*68:01, HLA-B*58:01 | **6IEX** |
| **16** | H-2-Lq | NPPIPVGEI | 253 | 261 | **HLA-B*51:01**, HLA-B*07:02, HLA-B*53:01, HLA-B*08:01, HLA-B*35:01, HLA-A*68:02, HLA-A*26:01, HLA-A*24:02, HLA-A*23:01, HLA-B*40:01, HLA-B*44:02, HLA-A*32:01 | **1E28** |
| **17** | H-2-Dd | QATQEVKNW | 308 | 316 | **HLA-B*58:01**, HLA-B*53:01, HLA-B*57:01, HLA-B*44:02, HLA-B*44:03, HLA-A*32:01, HLA-B*35:01, HLA-A*26:01, HLA-B*51:01, HLA-A*24:02, HLA-A*23:01, HLA-A*01:01, HLA-B*15:01, HLA-A*68:01, HLA-B*40:01, HLA-A*68:02, HLA-A*30:02, HLA-A*11:01, HLA-B*08:01, HLA-B*07:02, HLA-A*02:06 | **5VWH** |
| **18** | H-2-Dd | ANPDCKTIL | 326 | 334 | **HLA-B*40:01**, HLA-A*24:02, HLA-B*07:02, HLA-A*23:01, HLA-B*08:01, HLA-B*51:01, HLA-B*44:03, HLA-B*44:02, HLA-A*68:02 | **6IEX** |
| *** The bold font identified the Human HLA and the corresponding ID used in docking between epitopes and alleles.** | | | | | | |
