# Supplementary material for: Design of multivalent-epitope vaccine models directed toward the world’s population against HIV-Gag polyprotein: Reverse vaccinology and immunoinformatics
Source: PLoS One. 2024 Sep 27;19(9):e0306559. doi: 10.1371/journal.pone.0306559 (PMC11432917; doi:10.1371/journal.pone.0306559)
Supplement: S3 Table — (DOCX) [file pone.0306559.s003.docx]

**Table S3.** The techniques that were different in the development of vaccines in eukaryotic and prokaryotic hosts.

| **Technique** | **Eukaryotic host** | **Prokaryotic host** |
| --- | --- | --- |
| Cloning | pcDNA3.1(+) | pET-30a (+) |
| Codon optimization | Eukaryotic codon optimization system | Prokaryotic codon optimization system |
| HTL prediction tool | IEDB class II immunogenicity server | IEDB major histocompatibility complex (MHC) II binding server |
| Population coverage | Applicable for eukaryotic host | Not applicable |
