# Supplementary material for: Design of multivalent-epitope vaccine models directed toward the world’s population against HIV-Gag polyprotein: Reverse vaccinology and immunoinformatics
Source: PLoS One. 2024 Sep 27;19(9):e0306559. doi: 10.1371/journal.pone.0306559 (PMC11432917; doi:10.1371/journal.pone.0306559)
Supplement: S4 Table — (DOCX) [file pone.0306559.s004.docx]

**Table S4.** Accession numbers of 100 full-length HIV-1 sequences of the most predominant subtypes and CRFs

| **Accession Number** | **Subtype** | **Accession Number** | **Subtype** | **Accession Number** | **Subtype** |
| --- | --- | --- | --- | --- | --- |
| OQ121909.1 | G | OM209609.1 | B | MG760373.1 | AE |
| KX389646.1 | G | OM209714.1 | B | ON959789.1 | AE |
| KX228801.1 | G | OM209814.1 | B | OQ121842.1 | AG |
| JX245015.1 | O | OM209914.1 | B | MT417738.1 | AG |
| MH705150.1 | O | OM209937.1 | B | MH078539.1 | AG |
| AB703612.1 | 35-AD | OM209981.1 | B | KC473840.1 | 47-BF1 |
| OK649293.1 | C | MW881703.1 | B | MN090742.1 | B |
| OM825586.2 | C | MW881698.1 | B | MT222952.1 | B |
| OM825656.2 | C | JF320241.1 | B | MT417772.1 | B |
| OM825584.2 | C | JN692480.1 | B | ON421478.1 | B |
| AB254150.1 | C | JN692460.1 | B | ON421466.1 | B |
| AF443089.1 | C | JN692466.1 | B | ON421472.1 | B |
| AY463217.1 | C | JF320244.1 | B | MW881676.1 | B |
| AY253303.1 | C | JF320242.1 | B | MT417771.1 | B |
| OK649295.1 | C | OL446078.1 | B | MT417753.1 | B |
| OM825577.2 | C | MT559048.1 | B | ON421463.1 | B |
| AY713417.1 | C | MT559060.1 | B | ON421469.1 | B |
| AY772691.1 | C | OK514771.1 | B | MT417766.1 | B |
| AY878071.1 | C | MT559049.1 | B | MW881735.1 | B |
| DQ369982.1 | C | KR914676.1 | B | MN090736.1 | B |
| OM825578.2 | C | MZ642263.1 | A | MW262777.1 | B |
| MG365764.1 | F | MZ642262.1 | A | MW262770.1 | B |
| OM825109.2 | D | MZ642260.1 | A | ON816965.1 | B |
| OM825094.2 | D | MZ642266.1 | A | ON816970.1 | B |
| OM825104.2 | D | MZ642269.1 | A | OM209984.1 | B |
| MZ427710.1 | A6 | MT635148 | AE | MW881700.1 | B |
| MZ427733.1 | A6 | MT624753.1 | AE | MW881704.1 | B |
| OQ513524.1 | BC | AB253682.1 | AE | MW881706.1 | B |
| KF835547.1 | BC | ON902298.1 | AE | MW881707.1 | B |
| OQ092464.1 | B | MH327750.1 | AE | OM209730.1 | B |
| OQ092463.1 | B | ON816969.1 | B | OM209651.1 | B |
| OQ092465.1 | B | MW262776.1 | B | OM209404.1 | B |
| OQ092466.1 | B | OL519774.1 | B | OQ092462.1 | B |
| OQ092467.1 | B |  |  |  |  |
