# Supplementary material for: Design of multivalent-epitope vaccine models directed toward the world’s population against HIV-Gag polyprotein: Reverse vaccinology and immunoinformatics
Source: PLoS One. 2024 Sep 27;19(9):e0306559. doi: 10.1371/journal.pone.0306559 (PMC11432917; doi:10.1371/journal.pone.0306559)
Supplement: S5 Table — (DOCX) [file pone.0306559.s005.docx]

**Table S5.** ProtParam results for the HIV Gag protein and vaccine construct.

|  | **Number of amino acids** | **Molecular weight** | **Theoretical pI** | **Half-life in mammalian reticulocytes** | **Half-life in yeast** | **Half-life in *Escherichia coli*** | **Instability index** | **Aliphatic index** | **GRAVY** |
| --- | --- | --- | --- | --- | --- | --- | --- | --- | --- |
| **Gag Sequence** | 499 | 55743.73 | 9.27 | 30 hours | >20 hours | >10 hours | 44.88 | 70.60 | -0.644 |
| **Vaccine construct** | 585 | 61975.24 | 10.51 | 30 hours | >20 hours | >10 hours | 38.38 | 58.55 | -0.876 |
