# Supplementary material for: Design of multivalent-epitope vaccine models directed toward the world’s population against HIV-Gag polyprotein: Reverse vaccinology and immunoinformatics
Source: PLoS One. 2024 Sep 27;19(9):e0306559. doi: 10.1371/journal.pone.0306559 (PMC11432917; doi:10.1371/journal.pone.0306559)
Supplement: S6 Table — (DOCX) [file pone.0306559.s006.docx]

**Table S6.** The residue data and scores of the predicted linear BCL epitopes on the Gag vaccine construct.

| **No.** | **Chain** | **Start** | **End** | **Peptide** | **Number of residues** | **Score** |
| --- | --- | --- | --- | --- | --- | --- |
| **1** | A | 19 | 134 | AVLSCLPKEEQIGKCSTRGRKCCRRKKEAAAKAKFVAAWTLKAAAGGGSASVLSGGKLGGGSVLSGGKLDRGGGSIRLRPGGKKGGGSRLRPGGKKKGGGSKKYRLKHIVGGGSKY | 116 | 0.785 |
| **2** | A | 454 | 500 | RPKKRWEKIRLRPGGKKKYRKKGQLQPALQTGSEELKSKKQAAADTG | 47 | 0.769 |
| **3** | A | 351 | 405 | KYRLKHIGPGPGLRPGGKKKYRLKHIVGPGPGGKKKYRLKHIVWASRGPGPGYCV | 55 | 0.75 |
| **4** | A | 253 | 270 | DRLHPVGGGSNPPIPVGE | 18 | 0.676 |
| **5** | A | 527 | 557 | PKKFRFGEETTTPSQKQEPKKTTPSQKQEPI | 31 | 0.655 |
| **6** | A | 323 | 333 | IRLRPGGKKKY | 11 | 0.578 |
| **7** | A | 202 | 208 | VSQNYGG | 7 | 0.506 |
| **8** | A | 424 | 428 | PEVIP | 5 | 0.505 |
| **9** | A | 506 | 513 | SQNYKKEE | 8 | 0.505 |
