# Supplementary material for: Design of multivalent-epitope vaccine models directed toward the world’s population against HIV-Gag polyprotein: Reverse vaccinology and immunoinformatics
Source: PLoS One. 2024 Sep 27;19(9):e0306559. doi: 10.1371/journal.pone.0306559 (PMC11432917; doi:10.1371/journal.pone.0306559)
Supplement: S7 Table — (DOCX) [file pone.0306559.s007.docx]

**Table S7.** Population coverage results of selected CTL and HTL epitopes in the Gag vaccine construct

| **MHC Class I/II** | **Coverage** | **Average-hit** | **PC90** |
| --- | --- | --- | --- |
| Combined | 93.91 | 23.34 | 10.55 |
