# Supplementary material for: Design of multivalent-epitope vaccine models directed toward the world’s population against HIV-Gag polyprotein: Reverse vaccinology and immunoinformatics
Source: PLoS One. 2024 Sep 27;19(9):e0306559. doi: 10.1371/journal.pone.0306559 (PMC11432917; doi:10.1371/journal.pone.0306559)
Supplement: S8 Table — (DOCX) [file pone.0306559.s008.docx]

**Table S8.** Numbers of predicted CTL, HTL, and BCL epitopes

| **Gag gene** | **Primary epitopes** | **Final epitopes (common between human and mouse)** |
| --- | --- | --- |
| **CTL** | 5892 | 18 |
| **HTL** | 3888 | 7 |
| **BCL** | ABCpred=50, Bepipred=11, Emini= 494, Karplus=492, Parker=493  Total= 1545 | 7 |
