# Supplementary material for: Design of multivalent-epitope vaccine models directed toward the world’s population against HIV-Gag polyprotein: Reverse vaccinology and immunoinformatics
Source: PLoS One. 2024 Sep 27;19(9):e0306559. doi: 10.1371/journal.pone.0306559 (PMC11432917; doi:10.1371/journal.pone.0306559)
Supplement: S9 Table — The sequence of the vaccine model was antigenic, nontoxic, nonallergenic, and nonhomologous to the human proteome and lacked any signal sequence. (DOCX) [file pone.0306559.s009.docx]

**Table S9.** Results of screening the Gag vaccine construct. The sequence of the vaccine model was antigenic, nontoxic, nonallergenic, and nonhomologous to the human proteome and lacked any signal sequence.

|  | **Gag Vaccine** |
| --- | --- |
| **Toxin** | Non-Toxin |
| **Allergenicity** | NON-ALLERGEN |
| **VaxiJen** | 0.7128 (Probable Antigen) |
| **Topology** | Outside |
| **Homology** | Nonhomologous to human |
