# Supplementary material for: Design of multivalent-epitope vaccine models directed toward the world’s population against HIV-Gag polyprotein: Reverse vaccinology and immunoinformatics
Source: PLoS One. 2024 Sep 27;19(9):e0306559. doi: 10.1371/journal.pone.0306559 (PMC11432917; doi:10.1371/journal.pone.0306559)
Supplement: S10 Table — (DOCX) [file pone.0306559.s010.docx]

**Table S10.** The consensus sequences of the HIV-1 Gag gene and vaccine construct

|  | **Consensus of 100 sequences** |
| --- | --- |
| **Gag** | MGARASVLSGGKLDRWEKIRLRPGGKKKYRLKHIVWASRELERFAVNPGLLETSEGCRQILGQLQPALQTGSEELKSLYNTVATLYCVHQRIDVKDTKEALDKIEEEQNKSKKKAQQAAADTGNSSQVSQNYPIVQNLQGQMVHQAISPRTLNAWVKVIEEKAFSPEVIPMFSALSEGATPQDLNTMLNTVGGHQAAMQMLKETINEEAAEWDRLHPVHAGPIAPGQMREPRGSDIAGTTSTLQEQIGWMTSNPPIPVGEIYKRWIILGLNKIVRMYSPVSILDIRQGPKEPFRDYVDRFYKTLRAEQATQEVKNWMTETLLVQNANPDCKTILKALGPGATLEEMMTACQGVGGPGHKARVLAEAMSQVTNSNTIMMQRGNFRNQRKTVKCFNCGKEGHIARNCRAPRKKGCWKCGKEGHQMKDCTERQANFLGKIWPSHKGRPGNFLQSRPEPTAPPESFRFGEETTTPSQKQEPIDKELYPLASLKSLFGNDPSSQ |
| **Vaccine construct** | GGSVLSGGKLDRGGGSIRLRPGGKKGGGSRLRPGGKKKGGGSKKYRLKHIVGGGSKYRLKHIVWGGGSLKHIVWASRGGGSRFAVNPGLLGGGSATLYCVHQRGGGSTLYCVHQRIGGGSNSSQVSQNYGGGSHQAISPRTLGGGSSEGATPQDLGGGSATPQDLNTMGGGSAEWDRLHPVGGGSNPPIPVGEIGGGSQATQEVKNWGGGSANPDCKTILGPGPGKIRLRPGGKKKYRLKGPGPGIRLRPGGKKKYRLKHGPGPGRLRPGGKKKYRLKHIGPGPGLRPGGKKKYRLKHIVGPGPG GKKKYRLKHIVWASRGPGPGYCVHQRIDVKDTKEAGPGPGSPEVIPMFSALSEGAKKLSGGKLDRWEKIRLRPKKRWEKIRLRPGGKKKYRKKGQLQPALQTGSEELKSKKQAAADTGNSSQVSQNYKKEEAAEWDRLHPVHAGPKKFRFGEETTTPSQKQEPKKTTPSQKQEPIDKELYPEGGETAKSKKFPSYTATYQF |
