# Supplementary material for: Design of multivalent-epitope vaccine models directed toward the world’s population against HIV-Gag polyprotein: Reverse vaccinology and immunoinformatics
Source: PLoS One. 2024 Sep 27;19(9):e0306559. doi: 10.1371/journal.pone.0306559 (PMC11432917; doi:10.1371/journal.pone.0306559)
Supplement: S11 Table — (DOCX) [file pone.0306559.s011.docx]

**Table S11.** Various features of the secondary structures of the HIV Gag gene and vaccine construct

| **Groups and models** | **Alpha helix (Hh) (%)** | **Extended strand (Ee) (%)** | **Beta turn (Tt) (%)** | **Random coil (Cc) (%)** |
| --- | --- | --- | --- | --- |
| **Gag** | 45.49% | 7.62% | 4.41% | 42.48% |
| **Vaccine** | 29.74% | 15.21% | 6.50% | 48.55% |
