# Supplementary material for: Design of multivalent-epitope vaccine models directed toward the world’s population against HIV-Gag polyprotein: Reverse vaccinology and immunoinformatics
Source: PLoS One. 2024 Sep 27;19(9):e0306559. doi: 10.1371/journal.pone.0306559 (PMC11432917; doi:10.1371/journal.pone.0306559)
Supplement: S12 Table — (DOCX) [file pone.0306559.s012.docx]

**Table S12.** The residue data and scores of the predicted discontinuous BCL epitopes on the Gag vaccine construct.

| **No.** | **Residues** | **Number of residues** | **Score** |
| --- | --- | --- | --- |
| 1 | A:A19, A:V20, A:L21, A:S22, A:C23, A:L24, A:P25, A:K26, A:E27, A:E28, A:Q29, A:I30, A:G31, A:K32, A:C33, A:S34, A:T35, A:G37, A:R38, A:K39, A:C40, A:C41, A:K44, A:A250, A:D253, A:R254 | 26 | 0.841 |
| 2 | A:L82, A:S83, A:G84, A:G85, A:K86, A:L87, A:D88, A:R89, A:G90, A:G91, A:G92, A:S93, A:I94, A:R95, A:L96, A:R97, A:P98, A:G99, A:G100, A:K101, A:K102, A:G103, A:G104, A:G105, A:S106, A:R107, A:L108, A:P110, A:G111, A:G112, A:K113, A:K114, A:K115, A:G116, A:G117, A:G118, A:S119, A:K120, A:K121, A:R123, A:L124, A:K125, A:H126, A:I127, A:V128, A:G129, A:G130, A:G131, A:S132, A:K133, A:Y134, A:Q201, A:Y206, A:G207, A:G208, A:G209 | 56 | 0.748 |
| 3 | A:E535, A:T536, A:T537, A:T538, A:P539, A:S540, A:Q541, A:K542, A:Q543 | 9 | 0.743 |
| 4 | A:L325, A:R326, A:P327, A:G328, A:G329, A:K330, A:K331, A:K332, A:Y333, A:R353, A:K355, A:H356, A:I357, A:G358, A:P359, A:G360, A:P361, A:G362, A:L363, A:R364, A:P365, A:G366, A:G367, A:K368, A:K369, A:K370, A:Y371, A:R372, A:L373, A:K374, A:H375, A:I376, A:V377, A:G378, A:P379, A:G380, A:P381, A:G382, A:G383, A:K384, A:K385, A:K386, A:Y387, A:R388, A:L389, A:K390, A:H391, A:I392, A:V393, A:W394, A:A395, A:S396, A:R397, A:G398, A:P399, A:G400, A:P401, A:G402, A:Y403, A:C404, A:V405, A:H406, A:Q407 | 63 | 0.724 |
| 5 | A:I451, A:L453, A:R454, A:P455, A:K456, A:K457, A:R458, A:W459, A:E460, A:K461, A:I462, A:L464, A:R465, A:P466, A:G467, A:G468, A:K469, A:K470, A:K471, A:Y472, A:K474, A:K475, A:G476, A:Q477, A:L478, A:Q479, A:P480, A:A481, A:L482, A:Q483, A:T484, A:G485, A:S486, A:E487, A:E488, A:L489, A:K490, A:S491, A:K492, A:K493, A:Q494, A:A495, A:A496, A:A497, A:D498, A:T499, A:G500, A:S502, A:S506, A:N508, A:K510, A:K511, A:E512, A:E513, A:A515 | 55 | 0.715 |
| 6 | A:R42, A:R43, A:E46, A:A47, A:A48, A:A49, A:K50, A:A51, A:K52, A:F53, A:V54, A:A55, A:A56, A:W57, A:T58, A:L59, A:K60, A:A61, A:A62, A:A63, A:G64, A:G65, A:G66, A:S67, A:A68, A:S69, A:V70, A:L71, A:S72, A:G73, A:G74, A:K75, A:L76, A:G77, A:G78, A:G79, A:S80, A:V81, A:H256, A:P257, A:V258, A:G259, A:G260, A:G261, A:S262, A:N263, A:P264, A:P265, A:I266, A:P267, A:V268, A:G269, A:E270, A:G273, A:G274, A:S275, A:Q276, A:P291, A:D292, A:C293, A:K294, A:Q584 | 62 | 0.679 |
| 7 | A:S423, A:P424, A:E425, A:V426, A:I427, A:P428, A:E544, A:P545, A:K546, A:K547, A:T548, A:T549, A:P550, A:S551, A:Q552, A:K553, A:Q554, A:E555, A:P556, A:I557 | 20 | 0.639 |
| 8 | A:Q7, A:K8, A:Y10, A:R12 | 4 | 0.556 |
| 9 | A:R531, A:F532, A:G533, A:E534 | 4 | 0.532 |
