# Supplementary material for: Design of multivalent-epitope vaccine models directed toward the world’s population against HIV-Gag polyprotein: Reverse vaccinology and immunoinformatics
Source: PLoS One. 2024 Sep 27;19(9):e0306559. doi: 10.1371/journal.pone.0306559 (PMC11432917; doi:10.1371/journal.pone.0306559)
Supplement: S13 Table — (The best models are marked with a green box). (DOCX) [file pone.0306559.s013.docx]

**Table S13.** Molecular docking results of TLRs and the Gag vaccine construct generated using the Cluspro2.0 tool. (The best models are marked with a green box.)

|  | **Lowest energy scores** | | | | |
| --- | --- | --- | --- | --- | --- |
| **Predicted Models** | **Gag vaccine + TLR2** | **Gag vaccine + TLR3** | **Gag vaccine + TLR4** | **Gag vaccine + TLR7** | **Gag vaccine + TLR9** |
| **0** | -1020.2 | -1049.3 | -1194.8 | -1317.8 | -1184.4 |
| **1** | -1007.5 | -1088.5 | -1204.9 | -1099.5 | -1272.7 |
| **2** | -1072.8 | -1247.0 | -1192.3 | -1073.0 | -1118.5 |
| **3** | -1106.9 | -1044.0 | -1201.8 | -1220.9 | -1186.7 |
| **4** | -958.7 | -1062.6 | -1126.2 | -1130.8 | -1038.9 |
| **5** | -993.0 | -1100.2 | -1287.4 | -1140.4 | -1013.1 |
| **6** | -939.4 | -1336.9 | -1206.8 | -1180.5 | -1123.8 |
| **7** | -997.4 | -1218.8 | -1083.2 | -1020.6 | -1187.6 |
| **8** | -859.2 | -1036.9 | -1172.1 | -1020.4 | -1038.6 |
| **9** | -972.9 | -1133.4 | -1199.6 | -1012.5 | -1162.1 |
| **10** | -939.1 | -1105.7 | -1083.5 | -1085.6 | -985.8 |
| **11** | -935.5 | -1126.6 | -1102.5 | -1144.5 | -1146.4 |
| **12** | -908.5 | -989.7 | -1171.9 | -1101.7 | -1033.6 |
| **13** | -904.2 | -1103.1 | -1106.8 | -1004.5 | -1077.1 |
| **14** | -984.7 | -1049.5 | -1146.3 | -1018.4 | -1071.1 |
| **15** | -1016.0 | -1076.2 | -1114.5 | -1167.8 | -980.2 |
| **16** | -969.0 | -980.0 | -1099.9 | -1043.9 | -1006.6 |
| **17** | -978.3 | -927.1 | -1185.6 | -983.0 | -986.3 |
| **18** | -958.6 | -1022.0 | -1103.5 | -1105.3 | -997.0 |
| **19** | -973.4 | -1072.8 | -1039.9 | -1034.6 | -1016.1 |
| **20** | -953.9 | -972.6 | -1095.1 | -1065.4 | -1014.8 |
| **21** | -1053.0 | -1107.2 | -1218.2 | -1066.0 | -973.0 |
| **22** | -950.1 | -999.5 | -1020.4 | -1044.4 | -1045.7 |
| **23** | -927.5 | -994.3 | -1090.1 | -1115.3 | -969.0 |
| **24** | -1012.0 | -1015.3 | -1100.8 | -973.6 | -960.9 |
| **25** | -977.5 | -978.5 | -1206.6 | -934.9 | -950.9 |
| **26** | -932.9 | -1028.0 | -1162.1 | -986.2 | -1046.5 |
| **27** | -894.1 | -1048.5 | -1119.4 | -1024.4 | -999.4 |
| **28** | -929.8 | -921.7 | -1173.4 | -923.3 | -1055.3 |
| **29** | -931.0 | -968.8 | -1098.2 | -1056.0 | -972.9 |
