# Supplementary material for: Design of multivalent-epitope vaccine models directed toward the world’s population against HIV-Gag polyprotein: Reverse vaccinology and immunoinformatics
Source: PLoS One. 2024 Sep 27;19(9):e0306559. doi: 10.1371/journal.pone.0306559 (PMC11432917; doi:10.1371/journal.pone.0306559)
Supplement: S14 Table — (DOCX) [file pone.0306559.s014.docx]

**Table S14.** The sequence of the optimized codon of the vaccine models in human and *E. coli* hosts.

| **Hosts** | **Codon optimized sequences** |
| --- | --- |
| **Human** | GGAATCATTAACACCCTGCAGAAATACTACTGTAGGGTGCGCGGCGGTAGGTGCGCTGTGCTGTCCTGTCTGCCTAAGGAGGAACAGATCGGCAAATGCAGCACAAGAGGCAGAAAGTGCTGCAGGAGGAAGAAAGAGGCAGCCGCCAAGGCAAAATTCGTGGCCGCCTGGACTCTGAAGGCCGCCGCCGGCGGAGGGAGCGCCAGCGTGCTGTCCGGTGGAAAGCTCGGAGGGGGCTCTGTGCTGAGCGGAGGAAAACTGGACAGAGGAGGCGGGAGCATCAGGCTCCGCCCCGGCGGAAAGAAGGGCGGCGGCAGTCGGCTGCGGCCCGGCGGAAAAAAAAAAGGCGGGGGCTCCAAAAAGTACCGGCTGAAGCATATTGTGGGCGGAGGCTCTAAGTATAGGCTGAAGCACATCGTGTGGGGCGGCGGTAGCCTGAAACATATCGTGTGGGCCTCAAGGGGCGGAGGCTCCAGATTTGCCGTTAATCCAGGGCTGTTGGGAGGGGGATCAGCCACCCTGTATTGTGTGCACCAGCGCGGAGGCGGCTCCACACTCTACTGCGTGCACCAGCGGATTGGCGGTGGAAGTAACAGCTCTCAGGTGTCCCAGAATTATGGAGGCGGTAGCCATCAAGCCATCTCACCAAGAACCCTCGGCGGAGGCAGTTCTGAAGGCGCTACCCCCCAGGACCTGGGGGGTGGGAGTGCCACACCTCAAGACCTGAACACCATGGGCGGCGGATCAGCAGAATGGGACCGGCTGCACCCTGTTGGCGGCGGATCTAACCCTCCTATTCCCGTGGGCGAGATCGGCGGGGGCAGCCAGGCCACACAAGAAGTGAAAAACTGGGGCGGCGGCTCAGCCAACCCAGATTGCAAGACCATTCTGGGCCCCGGGCCCGGCAAGATTAGGCTGCGGCCTGGAGGAAAAAAGAAGTACAGGCTGAAGGGCCCTGGTCCAGGAATCAGACTGAGACCAGGCGGGAAGAAGAAGTACCGCCTGAAACATGGGCCAGGACCCGGCCGGCTGAGACCTGGAGGCAAGAAGAAATACCGGCTGAAGCATATCGGCCCTGGACCTGGCCTGCGGCCTGGCGGAAAGAAGAAGTACCGGCTGAAGCACATTGTGGGGCCAGGCCCCGGAGGGAAGAAGAAGTACCGCCTGAAACACATCGTGTGGGCCTCAAGGGGGCCAGGCCCTGGCTATTGCGTGCACCAGAGAATTGACGTGAAGGACACAAAAGAAGCTGGACCAGGCCCCGGCAGCCCCGAAGTGATTCCTATGTTCTCCGCCCTGAGCGAAGGCGCCAAAAAGCTGTCCGGCGGGAAGCTGGACAGGTGGGAGAAGATCAGACTGCGGCCAAAGAAGCGCTGGGAAAAAATTCGGCTGAGGCCTGGCGGAAAGAAGAAGTACAGGAAGAAGGGGCAGCTGCAGCCCGCCCTGCAGACAGGAAGTGAAGAGCTGAAATCTAAAAAACAGGCCGCTGCCGACACCGGAAATAGCAGCCAGGTGAGTCAGAATTACAAGAAAGAAGAGGCCGCTGAATGGGATCGCCTGCACCCCGTGCACGCTGGCCCTAAGAAGTTTAGGTTCGGCGAGGAGACCACAACTCCCTCACAGAAGCAGGAGCCCAAAAAGACAACCCCATCCCAGAAGCAGGAGCCAATTGACAAGGAACTGTACCCCGAGGGCGGCGAGACAGCCAAATCCAAGAAGTTCCCCTCCTATACCGCAACATACCAGTTCTGA |
| ***E. coli*** | GGCATTATTAATACGCTGCAGAAATATTATTGCCGTGTTCGCGGCGGCCGCTGCGCGGTGCTGAGCTGTCTGCCGAAAGAAGAACAGATTGGCAAATGCAGCACCCGTGGCCGCAAATGTTGTCGCCGTAAAAAAGAAGCGGCGGCGAAAGCCAAATTCGTGGCAGCGTGGACCCTGAAAGCGGCCGCGGGTGGCGGCAGCGCCAGCGTGCTGAGCGGCGGCAAACTGGGCGGCGGCAGCGTGCTGAGCGGCGGCAAACTGGATCGCGGCGGTGGCAGCATTCGTCTGCGTCCGGGCGGCAAAAAAGGCGGCGGTAGCCGCCTGCGCCCGGGCGGCAAAAAAAAAGGCGGCGGCAGTAAAAAATACCGCCTGAAACATATTGTGGGTGGCGGCAGCAAATATCGCCTGAAACATATTGTCTGGGGTGGCGGTAGCCTGAAACACATTGTGTGGGCGAGCCGTGGCGGCGGCAGCCGCTTCGCGGTGAACCCGGGCCTGCTGGGCGGCGGCAGCGCGACCCTGTACTGCGTGCATCAGCGCGGCGGCGGCAGCACCCTGTATTGCGTGCATCAGCGTATTGGCGGCGGCAGCAATAGCAGCCAGGTGAGCCAGAACTACGGTGGCGGTAGCCACCAGGCCATTAGCCCGCGTACCTTAGGCGGTGGCAGCAGCGAAGGTGCCACCCCGCAGGATCTGGGCGGCGGCAGCGCGACCCCGCAGGATCTGAATACCATGGGCGGTGGTAGCGCGGAATGGGATCGCCTGCATCCGGTGGGTGGCGGCTCCAACCCGCCGATTCCGGTTGGCGAAATTGGTGGCGGCAGCCAGGCGACCCAGGAAGTGAAAAATTGGGGAGGCGGCAGCGCGAACCCGGACTGTAAAACCATTCTGGGCCCGGGTCCGGGCAAAATCCGTCTGCGCCCGGGCGGCAAAAAAAAATACCGCCTGAAAGGCCCGGGCCCGGGCATTCGCCTGCGTCCGGGCGGCAAAAAAAAATATCGCCTGAAACATGGCCCGGGCCCGGGCCGTCTGCGTCCGGGCGGCAAAAAAAAGTATCGTCTGAAACATATTGGCCCGGGCCCGGGCCTGCGCCCGGGCGGCAAAAAAAAATATCGTCTGAAACATATTGTGGGCCCGGGCCCGGGCGGTAAAAAAAAATACCGCCTGAAACATATTGTATGGGCCAGCCGTGGTCCAGGCCCGGGCTATTGCGTGCATCAACGTATTGATGTTAAAGACACCAAAGAAGCGGGTCCGGGCCCGGGCTCACCGGAAGTGATTCCGATGTTCAGCGCCCTGTCGGAAGGCGCGAAAAAACTTTCGGGCGGCAAACTGGATCGCTGGGAAAAGATTCGCCTGCGCCCGAAAAAACGTTGGGAAAAAATTCGTCTGCGCCCGGGCGGCAAAAAAAAATATCGCAAAAAAGGCCAGCTGCAGCCGGCGCTGCAGACCGGCAGCGAAGAACTGAAAAGCAAAAAACAGGCGGCCGCGGATACCGGCAACAGCTCTCAGGTGAGCCAAAACTATAAAAAAGAAGAAGCGGCCGAATGGGATCGTCTTCACCCGGTCCACGCCGGCCCGAAAAAATTCCGCTTTGGTGAGGAAACCACCACCCCGTCCCAGAAACAGGAACCGAAAAAAACCACCCCGAGTCAGAAACAGGAACCGATTGATAAAGAACTGTATCCGGAAGGCGGCGAAACCGCGAAATCAAAGAAATTTCCGAGCTACACCGCGACCTATCAGTTTTAA |
