# Supplementary figures and images for: Design of multivalent-epitope vaccine models directed toward the world’s population against HIV-Gag polyprotein: Reverse vaccinology and immunoinformatics
Source: PLoS One. 2024 Sep 27;19(9):e0306559. doi: 10.1371/journal.pone.0306559 (PMC11432917; doi:10.1371/journal.pone.0306559)

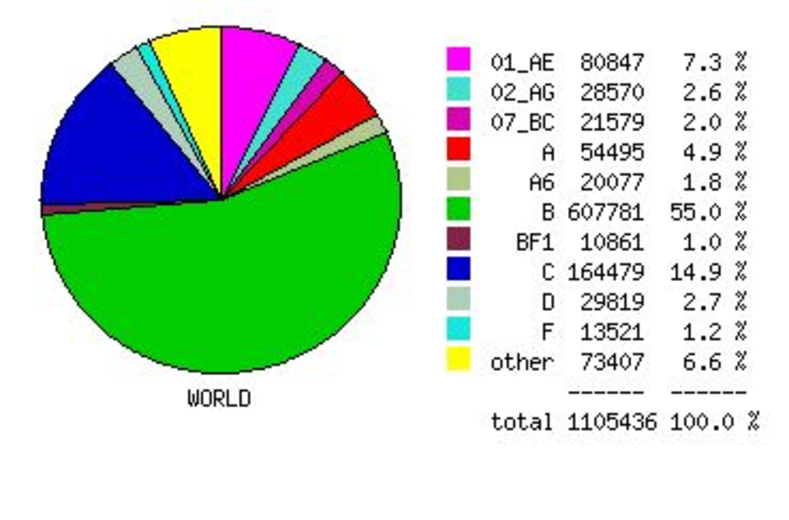

Supplement: S1 Fig — The figure was adjusted from the LANL database. (TIF) [file pone.0306559.s015.tif]
